# The combination of hydroxychloroquine and 2-deoxyglucose enhances apoptosis in breast cancer cells by blocking protective autophagy and sustaining endoplasmic reticulum stress

**DOI:** 10.1038/s41420-022-01074-6

**Published:** 2022-06-11

**Authors:** Ning Zhou, Qingyun Liu, Xiao Wang, Lixin He, Tao Zhang, Han Zhou, Xinying Zhu, Tianhong Zhou, Ganzhen Deng, Changwei Qiu

**Affiliations:** 1 Department of Clinical Veterinary Medicine, College of Veterinary Medicine, Huazhong Agricultural University, Wuhan, Hubei 430070 China; 2 State Key Laboratory of Agricultural Microbiology, College of Veterinary Medicine, Huazhong Agricultural University, Wuhan, Hubei 430070 China; 3 Department of Clinical Veterinary Medicine, College of Animal Science and Technology, Anhui Agricultural University, Hefei, 230036 China; 4 Department of Animal Science and Technology, Shanghai Agriculture and Forestry Vocational College, Shanghai, 201699 China

**Keywords:** Breast cancer, Drug development

## Abstract

2-Deoxyglucose (2-DG) can be used in antitumour research by inhibiting glycolysis and promoting the endoplasmic reticulum stress (ERS) pathway, but its clinical application is restricted due to dose-limiting side effects and survival chance for cancer cells by protective autophagy. Therefore, our research explored whether the combination of hydroxychloroquine (HCQ), an FDA-approved autophagy inhibiting drug, and 2-DG is a promising therapeutic strategy. Here, we report that HCQ combined with 2-DG can further inhibit the viability and migration and induce apoptosis of breast tumour cells compared with other individual drugs. The combination of 2-DG and HCQ can significantly reduce transplanted tumour size and tumour cell metastasis of the lung and liver in vivo. At the cellular level, HCQ suppressed autolysosome formation and terminated the autophagy process induced by 2-DG-mediated ERS, resulting in the continuous accumulation of misfolded proteins in the endoplasmic reticulum, which generated sustained ERS through the PERK-eIF2α-ATF-4-CHOP axis and triggered the transformation from a survival process to cell death. Our research reinforced the research interest of metabolic disruptors in triple-negative breast cancer and emphasized the potential of the combination of 2-DG and HCQ as an anticancerous treatment.

## Introduction

Breast cancer is one of the most commonly diagnosed tumours in women [1]. Despite the clinical application of new techniques such as early detection, imaging and targeted therapy, a large number of women die each year of breast cancer progression [2]. Canine breast tumours (CMTs) are also one of the high incidence types of tumours in unneutered dogs [3]. CMTs are one of the best animal models for studying human tumours because they share similar physiological, genetic, and epidemiological characteristics [4]. However, the currently used chemotherapy drugs are limited due to their strong cytotoxic side effects and drug resistance [5, 6]. Thus, safe and effective novel therapeutic strategies are urgently needed.

Cancer cells are more likely to obtain energy from glucose by glycolysis than normal cells, which produce ATP through oxidative phosphorylation [7]. Therefore, the suppression of glycolysis is considered an effective potential therapeutic strategy for breast tumours [8].

2-Deoxyglucose (2-DG) is a glucose analogue, and it can bind to glucose transporters, which are key proteins for glucose absorption, to competitively inhibit glucose absorption and further inhibit glycolysis [9, 10]. In addition, 2-DG-GDP is a downstream product of 2-DG metabolism, which can interfere with N-linked glycosylation due to its similarity to mannose-GDP in structure [11]. Therefore, 2-DG can also induce the continuous accumulation of unfolded/misfolded proteins in the endoplasmic reticulum (ER), which could cause endoplasmic reticulum stress (ERS) and subsequent death [12, 13]. On the other hand, ERS activates protective autophagy and mediates the elimination of defective proteins and organelles, which may be the reason why 2-DG monotherapy has yielded few positive results [14, 15].

Hydroxychloroquine (HCQ) is widely used in the treatment of clinical diseases, including rheumatology and infectious diseases [16, 17]. Moreover, recent research evidence shows the positive role of HCQ in oncology research because it can inhibit autophagy by deacidifying lysosomes [18, 19].

In this study, we show that the combined treatment of HCQ and 2-DG has a synergistic effect on inhibiting the viability of 4T1 cells (mouse origin) and CMT-7364 cells (canine origin) under the mechanism of autophagy inhibition and sustained ERS.

## Results

### HCQ and 2‐DG display cytotoxicity on breast cancer cells

**Fig. 1:**
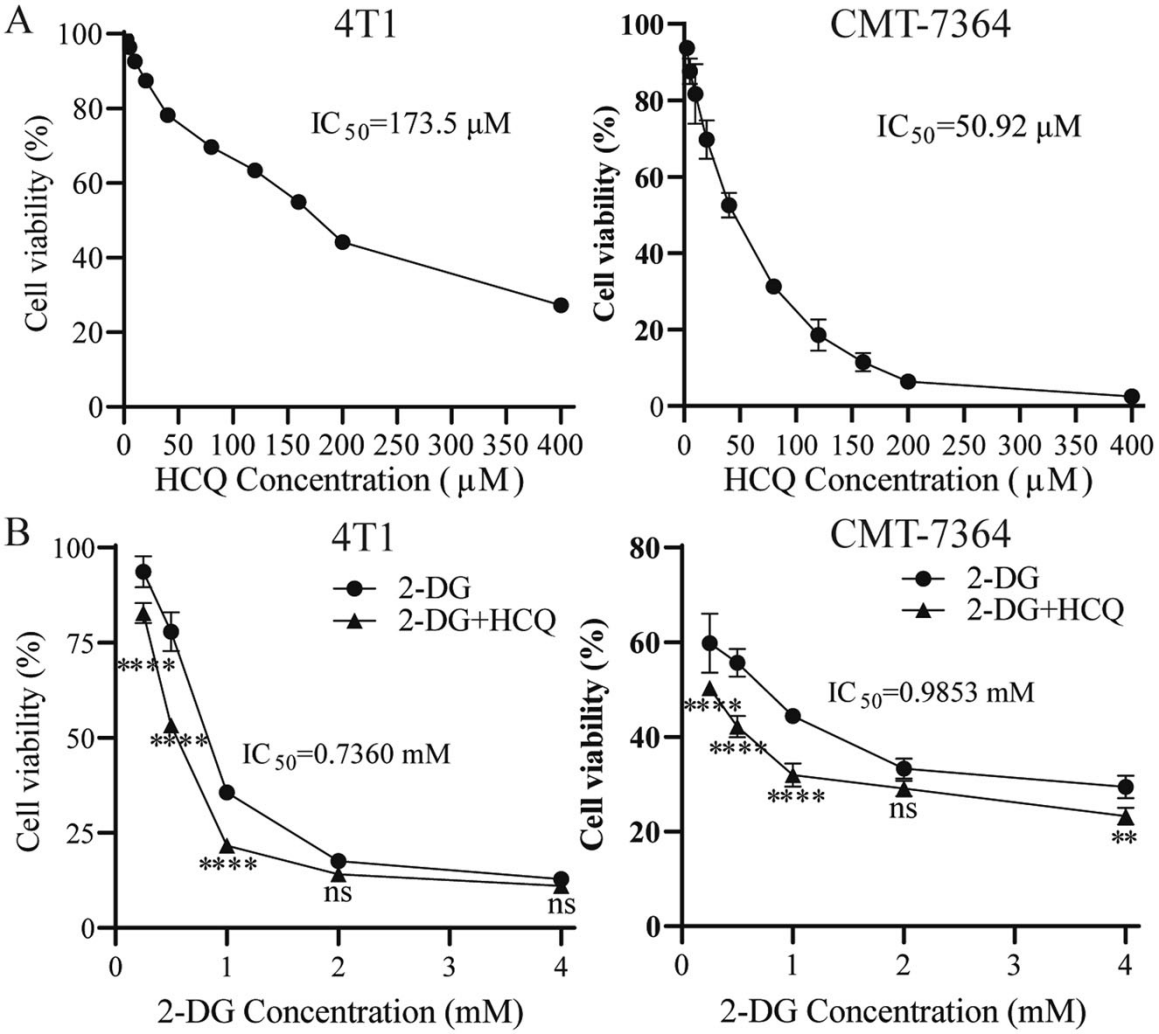
HCQ and 2‐DG inhibit breast cancer cell viability. **A**, **B** The 24 h IC50 values of HCQ and 2‐DG were calculated in 4T1 and CMT-7364 cells. Cell viability was determined via the Cell Counting Kit‐8. The combination of 2-DG and HCQ had a stronger cytotoxic effect on cell viability than 2-DG alone. *n* = 5 for each group. Data are presented as the means ± SD of three independent experiments. **p < 0.01, ****p < 0.0001, ns = not significant (*p* ≥ 0.05).

As previously reported, HCQ and 2‐DG are cytotoxic to a variety of cancer cells [12, 19]. To explore the mode of action of drugs in different species of breast tumours more comprehensively, we chose CMT-7364 and 4T1 cells, which are all usually applied models of human breast tumours. Cells treated with increasing concentrations of HCQ or 2‐DG showed a dose-dependent decrease in viability (Fig. 1A, B). The IC50 values of CMT-7364 and 4T1 cells treated with HCQ were both >50 μM. Based on these results and previous reports, we chose 10 μM HCQ for further studies. In contrast to 2-DG alone, 10 μM HCQ combined with different concentrations of 2-DG increased cytotoxicity (Fig. 1B). The IC50 of 2-DG in CMT-7364 and 4T1 cells was approximately equal at 0.9853 and 0.7360 mM, respectively. Based on these findings, 1 mM 2-DG was used for further experiments.

### HCQ combined with 2-DG exerts a higher anticancer activity in vitro

**Fig. 2:**
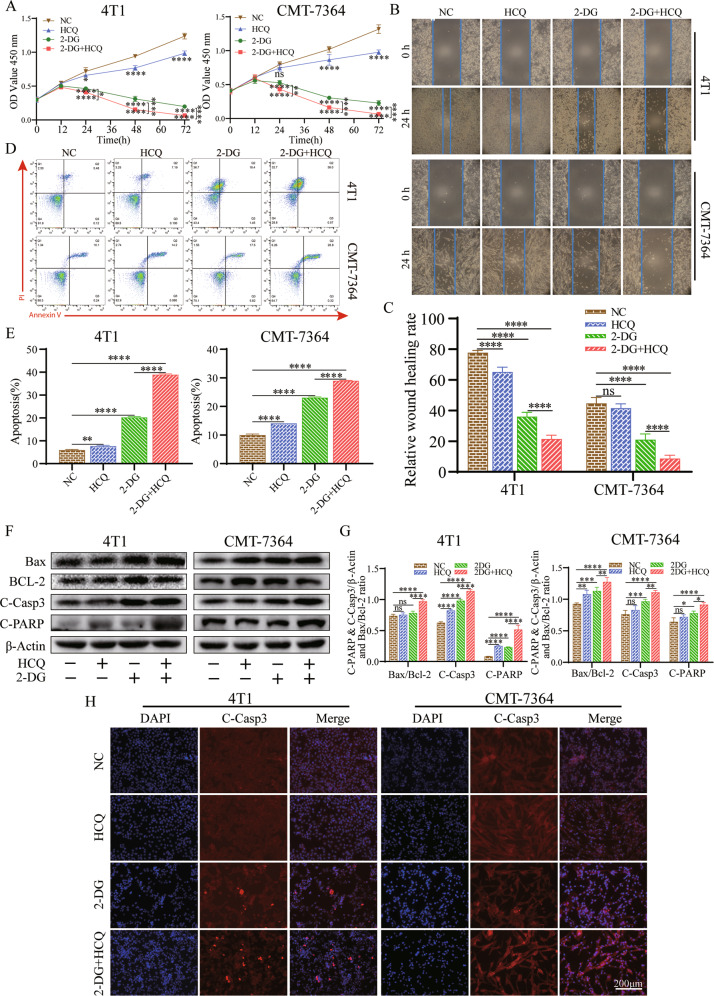
HCQ synergizes with 2‐DG to decrease cell proliferation and promote apoptosis in 4T1 and CMT-7364 cells. **A** Cell Counting Kit‐8 kits were used to assess the proliferation of 4T1 and CMT-7364 cells treated with HCQ, 2‐DG or HCQ combined with 2‐DG at 0, 12, 24, 48 and 72 h. *n* = 3 independent experiments performed in quintuplicate for each condition. **B**, **C** Wound-healing assays of 4T1 and CMT-7364 cells after HCQ and 2‐DG treatment alone or in combination for 24 h. Representative images depicting the beginning (*t* = 0 h) and the end (*t* = 24 h) of the recording period are shown. *n* = 3 independent experiments performed in triplicate for each condition. **D**, **E** Cell apoptosis assays of 4T1 and CMT-7364 cells treated with HCQ, 2‐DG or their combination using FACS. Cells were collected and labelled with Annexin V‐FITC and PI. *n* = 3 independent experiments performed in triplicate for each condition. **F**, **G** Western blot analyses of Bax, Bcl‐2, cleaved caspase-3 (C-Casp3), and cleaved PARP (C-PARP) in cells treated with HCQ and 2‐DG alone or in combination for 24 h. β‐actin was used as an internal control. **H** Immunofluorescence staining of C-Casp3 in 4T1 and CMT-7364 cells, which were treated with HCQ and 2‐DG alone or in combination for 24 h. Scale bar: 200 μm. *n* = 3 independent experiments performed in triplicate for each condition. All results from three independent experiments are expressed as the mean ± SD. *p < 0.05, **p < 0.01, ***p < 0.001, ****p < 0.0001, ns = not significant (*p* ≥ 0.05).

2-DG has been shown to inhibit cell proliferation and induce cell apoptosis in breast neoplasms but usually needs to be combined with other drugs to obtain better results [19, 20]. Compared with the NC group, treating with different concentrations of 2-DG all significantly increased the expression of apoptotic proteins in CMT-7364 cells (Figure S1). While, there was no difference in these protein expression levels between the different concentration groups (Figure S1), suggesting the dose‐limiting side effects of 2-DG in CMT-7364 cells. Therefore, we explored whether the addition of HCQ can reduce the effective drug dose of 2-DG in CMT-7364 and 4T1 cells. Compared to the NC group, the viability of CMT-7364 and 4T1 cells at 48 and 72 h following treatment with HCQ and 2-DG was significantly inhibited, and their combination treatment resulted in lower cell viability and cell density than 2-DG alone (Fig. 2A). This phenomenon suggests massive cell death occurred.

A wound-healing assay was used to investigate the relationship between the decrease in cell viability and the inhibition of cell migration. We did not observe the effect of HCQ alone, whereas 2-DG and their combination significantly inhibited cell migration in CMT-7364 cells. In contrast, HCQ and 2-DG alone similarly reduced cell migration compared with the control, and their combination significantly inhibited cell migration in 4T1 cells (Fig. 2B, C). These results indicate that the combination of HCQ and 2-DG strongly suppressed cell migration effects.

Apoptosis is an essential regulatory process that maintains the cellular homoeostasis of cancer cells. Therefore, the apoptotic rates were measured by Annexin V/PI staining. The results indicated that the apoptosis of the HCQ and 2-DG cotreatment group was significantly higher than that of the negative control and individual treatment groups (Fig. 2D, E). In addition, we also evaluated the protein levels of Bax, Bcl‐2, C-Caspase-3 and C-PARP, which are typical apoptotic proteins in cancer cells (Fig. 2F, G). The expression levels of C-Caspase-3 proteins were detected by immunohistochemical analysis (Fig. 2H). These results are consistent with the above conclusions. Altogether, these results suggest that the combination of 2-DG and HCQ contributes to effective antitumour properties in vitro.

### HCQ and 2-DG suppressed 4T1 xenograft tumour growth in vivo

**Fig. 3:**
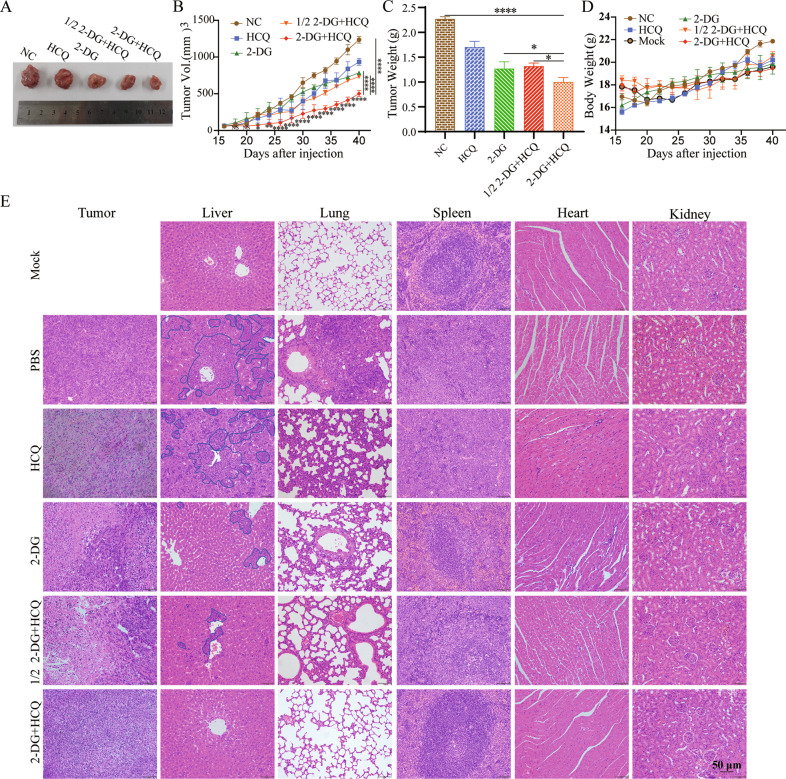
In vivo, HCQ combined with 2‐DG inhibits xenograft tumour growth. BALB/c female mice were divided into five groups, and injection of the indicated drugs began on the fifteenth. **A**–**C** Every 2 days, the tumour volume and body weight were measured (*n* = 5 per group). **D** On Day 40, the mice were humanely killed, samples were collected, and the tumour weight was measured. Data are shown as the mean ± SD. The symbols ns, *, **, **** denote significant differences of not significant (*p* ≥ 0.05), *P* < 0.05, *P* < 0.01, *P* < 0.0001 versus the negative control group, respectively. **E** Representative panels of H&E staining of tumours and major vital organs, such as the liver, lung, spleen, heart and kidneys of mice. Scale bar: 50 μm. Blue-lined bordered boxes at the sections of the liver indicate metastatic 4T1 cells.

To further validate the results of the in vitro experiments, a subcutaneous homotransplant mouse model was established. As the results show (Fig. 3A–C), the tumour volumes and weights of the combined treatment of HCQ and 2-DG were significantly lower than those of the negative control after 4 weeks of treatment. The results of 2-DG and 1/2 2-DG combined with HCQ treatment showed similar curves. No significant difference in body weight was encountered by the treated mice compared with the controls (Fig. 3D). We further examined the histopathological features of tumours and vital organs by H&E staining (Fig. 3E). No evident histopathological abnormalities were observed in the spleen, heart or kidney, showing little-to-no toxic and side effects of the drugs on mice. Tumour sections of the combination group showed increased proportion of cells with fragmented nuclei than those of 2-DG group, HCQ group and NC group, illustrating severer pathological changes of the tumour in situ treated with 2-DG and HCQ. In addition, the degree of nuclear fragmentation in tumour cells was similar in 100 mg/kg 2-DG group and 50 mg/kg 2-DG combined with HCQ group, suggesting the dose reduction of 2-DG by the addition of HCQ. These tumour section changes were consistent with the changes of tumour volume and weight in Fig. 3A–C.

Moreover, significant tumour metastasis was observed in the liver and lung of the PBS injection control group (the metastasis was indicated by the blue line boxes in the liver section and by the cells filled between the alveoli in the lung section). Compared with the tumour metastasis in the single-drug group and the NC group, the metastatic foci in the liver and lung were significantly reduced in 2-DG and HCQ group, suggesting that the combination of drugs might reduce the tumour metastasis. However, this result needs to be supported by more experimental data, which may be the emphasis of follow-up research on the combination of 2-DG and HCQ.

Furthermore, immunofluorescence staining of the tumour sections showed that the accumulation of LC3 and Cleaved-Caspase-3 obviously increased in the 2-DG and HCQ combination treatment group, compared with NC group, HCQ group and 2-DG group (Figure S2). Therefore, it was suggested that the combination of HCQ and 2-DG could enhance the autophagy and apoptosis in 4T1 cells. Moreover, the fluorescence intensities of LC3 and Cleaved-Caspase-3 in cells treated with 50 mg/kg 2-DG combined with HCQ were similar to those in cells treated with only 100 mg/kg 2-DG, revealing that the addition of HCQ could reduce the effective concentration of 2-DG (Figure S2). Together, these observations supported that HCQ and 2‐DG combination treatment exhibits stronger anticancer activity in vivo.

### The HCQ and 2-DG combination treatment induces ERS via the PERK/eIF2α/ATF-4/CHOP signalling pathway

**Fig. 4:**
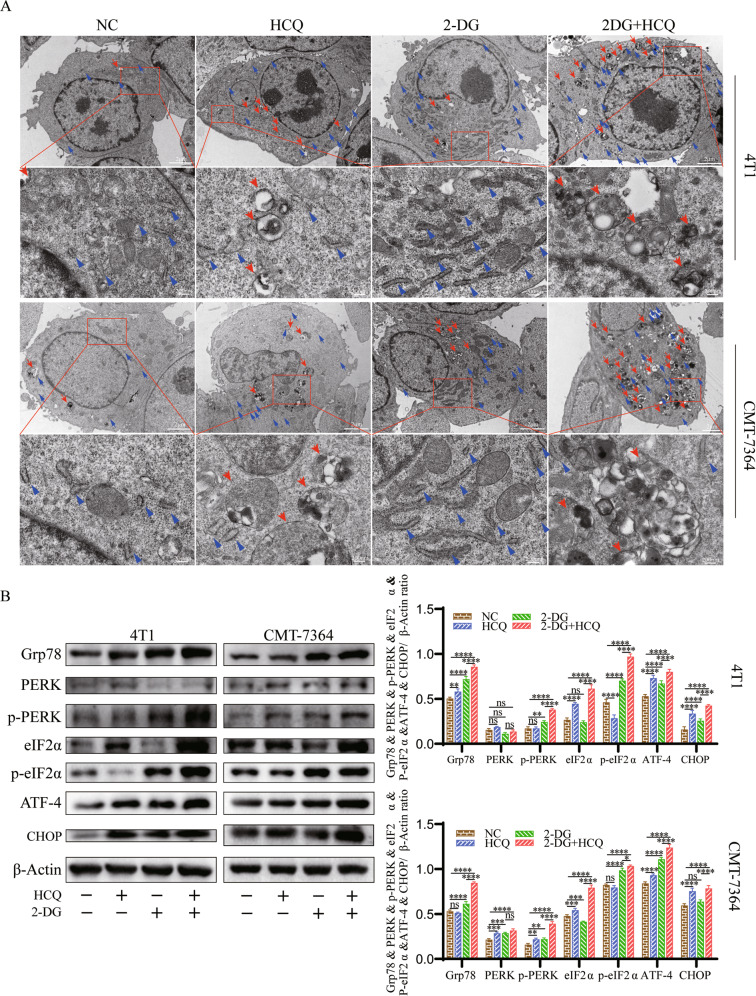
HCQ and 2-DG synergistically promote endoplasmic reticulum stress through the PERK/eIF2α/ATF-4 pathway. **A** Representative TEM images depicting the ultrastructure of 4T1 and CMT-7364 cells treated without or with HCQ, 2-DG or 2-DG combined with HCQ for 24 h. Red arrows indicate autophagic vacuoles; blue arrows indicate endoplasmic reticulum. Scale bars: 2 mm (Thumbnail), 200 nm (Enlarge image). **B** Western blot analyses of Grp78, PERK, p-PERK, eIF2α, p-eIF2α, ATF-4 and CHOP in cells treated with HCQ and 2‐DG alone or in combination for 24 h. β‐actin was used as an internal control. Data are represented as mean ± SD from three independent experiments. *p < 0.05, **p < 0.01, ***p < 0.001, ****p < 0.0001, ns = not significant (*p* ≥ 0.05).

To further investigate the enhanced anticancer effects of the combination of HCQ and 2-DG, transmission electron microscopy (TEM) was used to observe the cell morphology. Quantitative statistical analysis of the number of ER and autophagic vesicles is shown in Fig. S3. After treatment with 2-DG, the electron density, number and length of the ER in 4T1 and CMT-7364 cells increased overall. This phenomenon is in line with previous research [21]. However, after the combined treatment of HCQ and 2-DG, the length of ER of 4T1 and CMT-7364 cells was shortened to varying degrees, while the electron density and quantity barely changed (Fig. 4A). This discovery prompted us to investigate the unfolded protein response (UPR) pathways, while the PERK/eIF2α/ATF-4/CHOP pathway is the major pathway in anaphase of ERS and can directly induce apoptosis [22, 23]. The cotreatment group dramatically induced the expression of Grp78, p-PERK, p-eIf‐2α, ATF-4 and CHOP compared with the NC group and the treatment group alone (Fig. 4B). Overall, the HCQ and 2‐DG combination induces ERS by activating the PERK/eIF2α/ATF-4/CHOP pathway in 4T1 cells and CMT-7364 cells.

### Combination treatment with HCQ and 2-DG induces autophagy, the accumulation of which occurs through blockade of autophagic flux with HCQ

**Fig. 5:**
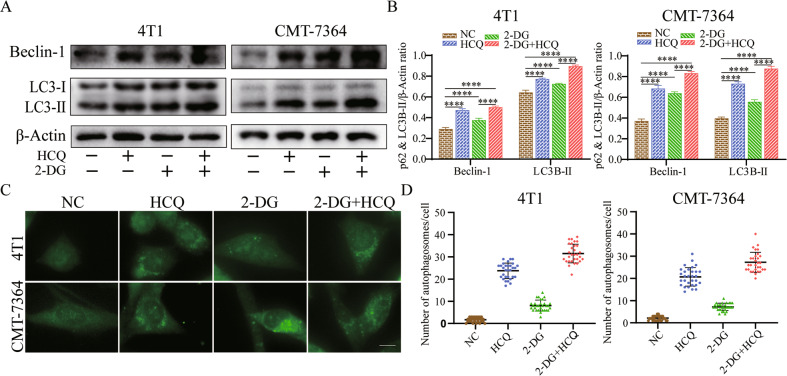
HCQ combined with 2-DG inhibits autophagic degradation in 4T1 cells and CMT-7364 cells. **A**, **B** Western blot analyses of Beclin-1 and LC3B-II in 4T1 and CMT-7364 cells that were treated without or with HCQ, 2-DG or 2-DG combined with HCQ for 24 h. β‐actin was used as an internal control. Data are represented as mean ± SD from three independent experiments. **p < 0.01, ****p < 0.0001. **C**, **D** Immunofluorescence staining of LC3B-II in 4T1 and CMT-7364 cells, which were treated with HCQ and 2‐DG alone or in combination for 24 h. Scale bar: 10 μm. *n* = 3 independent experiments performed in triplicate for each condition and at least 30 cells scored.

HCQ can increase the pH of lysosomes, which leads to inhibition of the fusion of lysosomes and autophagosomes and blocks autophagic flux [24, 25]. As the TEM analysis showed (Fig. 4A), the combination therapy significantly promoted an increase in autophagic vacuoles. Then, Western blot analysis was utilized to detect the expression of the autophagic markers Beclin-1, LC3B-I/II in 4T1 and CMT-7364 cells. As shown in the quantified results (Fig. 5A, B), the cotreatment group dramatically increased Beclin-1 levels and LC3B lipidation, while the images of fluorescent LC3B puncta also confirmed this result (Fig. 5C, D).

## Discussion

Triple-negative breast tumour (TNBC) is a collective term for breast tumour subtypes that lack the oestrogen receptor and progesterone receptor and do not overexpress human epidermal growth factor receptor 2 [26]. Compared with tumours that positively express oestrogen receptor, progesterone receptor, and human epidermal growth factor receptor 2, TNBC is more aggressive and has worse treatment effects and prognosis [27]. CMT-7364 (canine origin) is a type of TNBC cell with similar invasive and transfer characteristics to MDA-MB-231 cells (human origin) [28]. For better comparison, we also studied 4T1 cells (mouse origin), which also have the semblable characteristics of TNBC [29].

**Fig. 6:**
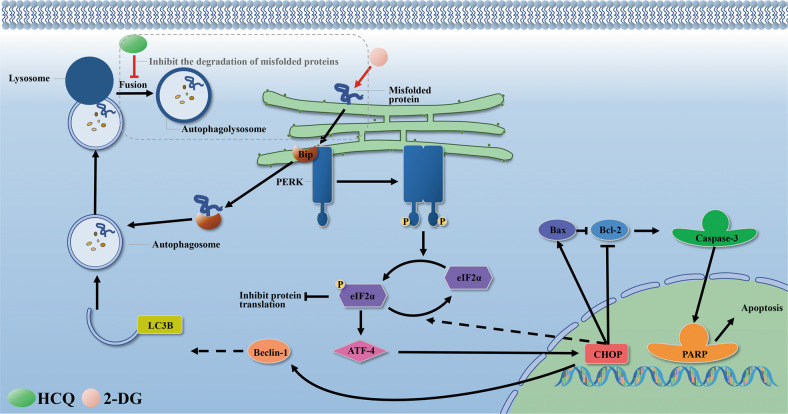
Schematic illustration delineating the role of HCQ/2‐DG combination therapy. The metabolites of 2-DG interfere with the N-linked glycosylation of proteins, leading to the accumulation of unfolded/misfolded proteins in the ER, followed by ERS, apoptosis, and cytoprotective autophagy. HCQ causes deacidification of lysosomes, inhibits the fusion of autophagosomes and lysosomes to form autolysates, which results in the inhibition of misfolded protein degradation and further induces endoplasmic reticulum stress, ultimately leading to increased apoptosis.

2-DG, a glycolysis inhibitor, has been demonstrated to induce apoptosis in cancer cells by inhibiting glycolysis and energy production and activating the ERS pathway [13, 30, 31]. However, only the high-dose treatment of 2-DG can achieve good results for practical applications, which restricts the generalization of 2-DG to a certain extent. HCQ can cause an elevation of pH in lysosomes, which can inhibit the fusion of autophagosomes and autolysosomes and the degradation of cargo in autophagosomes [32]. Here, we show that the combined treatment of 2-DG and HCQ leads to an increase in cell apoptosis by inducing ERS and inhibiting autophagy (Fig. 6).

The transcription Factor C/EBP homologous protein (CHOP) is involved in ERS, which can regulate apoptosis through the BCL-2 protein family [33]. Under persistent ERS, CHOP can inhibit the expression of Bcl-2, Bcl-XL and Mcl-1, activate the expression of BIM and induce an increase in BAX and BAK [34]. This regulation initiates the activation of caspase-3 and PARP and leads to apoptosis [35]. In our research, we found that HCQ and 2-DG both inhibited the activity of CMT-7364 and 4T1 cells in a gradient concentration-dependent and time-dependent manner, and the combined use of HCQ and 2-DG further enhanced the inhibitory effect (Fig. 1). Similar outcomes were observed in the rate of migration and apoptosis in TNBC cells. The detection of apoptotic proteins also illustrates this point (Fig. 2). In orthotopic transplantation tumour mouse models, the therapeutic effect of 100 mg/kg 2-DG used alone was similar to that of 50 mg/kg 2-DG combined with HCQ group, both of which reduced tumour volume and weight to a certain extent (Fig. 3, Figure S2). Furthermore, the 2-DG combined with HCQ group presented a more significant therapeutic effect than the other groups (Figs. 3, S2). These results together illustrated that cotreatment with 2-DG and HCQ could effectively promote apoptosis by inhibiting autophagy to inhibit the tumour growth.

The ER is the most important organelle in the secretory function of eukaryotic cells and is responsible for the storage of calcium ions, the metabolism of lipids and carbohydrates, and the folding of proteins. Due to the special growth characteristics and environment of tumour cells, many stimulating factors, including hypoxia, lack of amino acids, and insufficient glucose supply, will affect protein processing in the ER. ERS refers to an increase in unfolded or misfolded proteins caused in the ER. To maintain cell homoeostasis, cells initiate multiple basic pathways, including the UPR, endoplasmic reticulum-related degradation, autophagy, etc. Whether these regulatory programmes are sufficient to maintain cell function determines whether the cell is to restore a steady state or activate the cell death programme [36–39]. The UPR signalling pathway consists of three major branches: protein kinase R-like ER kinase (PERK), inositol requiring enzyme 1 (IRE1) and activating transcription factor 6 (ATF6). Studies have pointed out that at the beginning of the UPR response, all branches are rapidly activated for cytoprotection. As the ERS continues, the IRE1 and ATF6 signals will be downregulated, creating an imbalance, while the PERK signal will continue to output and guide the cell towards its demise [40, 41]. Therefore, the PERK pathway was the focus of this study, as with other studies [42, 43]. The microscopic characterization of organelles was observed by TEM. The statistical analysis results of TEM are shown in Fig. S3. We found that the number of widened ERs was significantly increased in TNBC cells treated with HCQ. However, after 2-DG treatment, the number, electron density and length of ER in cells all increased significantly compared with NC. Interestingly, ER was significantly shorter in the cotreatment group, but its number and electron density remained at a high level. We hypothesized that the ER, as the main source of the intracellular plasma membrane, provides membrane sources for the massively formed autophagic vesicles, thereby shortening the length of its own plasma membrane [44, 45]. WB results showed that HCQ combined with 2-DG significantly increased ERS levels through the PERK-eIF2α-ATF-4-CHOP pathway (Fig. 4).

From yeast to mammals, autophagy is an evolutionarily conserved process [46, 47]. A high degree of autophagy has been found in many tumour cells to maintain cell survival [48, 49]. Autophagy participates in the degradation of damaged proteins/organelles in cells and converts them into amino acids [50]. These findings support the importance of autophagy in sustaining cellular homoeostasis. TEM showed an increase in autophagic vesicles in TNBC cells after 2-DG treatment compared with NC. Previous studies have shown that 2-DG induces the formation of autophagosomes to enhance autophagy [51]. Combined with our study, it is speculated that 2-DG might induce protective autophagy response by glucose deprivation [52–55]. Therefore, blocking protective autophagy has become the subject of many studies [56–58]. HCQ was applied to block the protective autophagy in our study. In TNBC cells treated with a combination of 2-DG and HCQ, the number of autophagic vesicles and the protein levels of Beclin-1 and LC3B-II increased significantly than those treated with the single-drug groups (Figs. 4 and 5). It is indicated that HCQ terminated the protective autophagy induced by 2-DG.

In this study, we demonstrated the effective antitumoral effect of the combination of 2-DG and HCQ on TNBC cells both in vivo and in vitro. Indeed, the remarkable efficiency of their combination to affect cell viability by autophagy inhibition and continuous ERS may have important implications in the treatment of breast cancer.

## Materials and methods

### Cell culture

The 4T1 cells used in this study were obtained from the Chinese Academy of Sciences Cell Bank (Shanghai, China), and CMT-7364 cells, a cell line isolated from canine breast tumour, were kindly provided by the Degui Lin Laboratory at the China Agricultural University [59]. All cell lines were grown in RPMI 1640 medium with 10% foetal bovine serum and 1% penicillin–streptomycin at 37 °C with 5% CO_2_. Cells were cultured in T25 cell culture flasks. When the cell density reached more than 80%, the cells were subcultured into 96‐well plates or 6‐well plates for the corresponding experiments.

### Reagents and antibodies

Western blotting and immunofluorescence were performed using the following antibodies: phospho‐PERK (p-PERK), phospho‐eIF2α (p-eIF2α), cleaved‐Caspase-3 (c-Casp3), β‐actin (Cell Signalling Technologies; Danvers, MA); cleaved‐PARP (c-PARP), CHOP, Bax, Bcl‐2, Grp78, PERK, eIF2α, ATF-4, Beclin-1, LC3B-I/II (Abcam, Cambridge, UK). 2‐DG (purity ≥98%) and HCQ (purity >99%) were obtained from Sigma‐Aldrich Chemical (Agent of Shanghai, China). The Apoptosis Assay Kit and FITC Annexin V Apoptosis Detection Kit with PI (propidium iodide) were purchased from the Beyotime Institute of Biotechnology (Shanghai, China). The Cell Counting Kit‐8 was purchased for cell viability detection (CCK‐8, Dojindo Laboratories, Minato‐ku, Tokyo, Japan). All of the other chemicals and reagents were of the highest commercial grade availability.

### Cell viability (CCK-8) assay

Cells were seeded in 96‐well plates at a density of 2 × 10^4^ cells/mL (37 °C, 12 h). There were five repeats for one group, and different concentrations of 2-DG (0.25, 0.5, 1, 2 or 4 mM) combined with or without HCQ (10 μM) and HCQ (5, 10, 20, 40, 80, 120, 160, 200 or 400 μM) were added and incubated for another 24 h. In the other set of experiments, cells were cultured with PBS, HCQ (10 μM), 2-DG (1 mM), or 2-DG (1 mM) combined with HCQ (10 μM) for different times (12, 24, 48 or 72 h). Culture medium with the same concentration of drugs was changed every 12 h unless otherwise indicated. After treatment, a total of 10 µL (5 mg/mL) of CCK-8 was added per well and incubated at 37 °C for 1 h. Cell viability was measured through absorbance (optical density) with a microplate reader (Bio‐Rad Instruments, Hercules, CA) at 450 nm.

### Wound-healing migration assay

Cell migration was assessed by wound-healing scratch assays as previously described. CMT-7364 cells and 4T1 cells were seeded into 6-well tissue culture plates. When cells grew to 80% confluence, scratches were made using 200 μL plastic pipette tips, and the cells were washed twice with PBS carefully. Then, the cells were stimulated with Opti-MEM (Invitrogen, CA) reduced serum medium containing PBS, HCQ, 2-DG or 2-DG combined with HCQ for 24 h. Images were taken from an inverted microscope connected to a Canon camera. ImageJ software was used to measure the gap distance of the wound.

### Apoptosis assay

Apoptosis assays were performed using Annexin V-FITC (fluorescein isothiocyanate) and propidium iodide (PI) double staining. CMT-7364 cells and 4T1 cells were cultured in 6-well plates for 12 h. After the predetermined treatment was reached, the cells were washed twice with precooled PBS. The cells were digested with trypsin and centrifuged at a speed of 1000 rpm/min for 5 min, washed twice with precooled PBS, and resuspended in binding buffer (500 μL) at a concentration of 1 × 10^6^ cells/mL. The cells were gently vortexed and incubated with 5 μL of Annexin V-FITC and 10 μL of propidium iodide at room temperature for 10 min in the dark per the manufacturer’s instructions. The apoptosis rates were determined by flow cytometry (Becton Dickinson; Franklin Lakes, New Jersey) and analysed by FlowJo 10.6.2 software.

### Transmission electron microscopy

CMT-7364 cells and 4T1 cells were cultured in 6-well plates. After treatment, cells were fixed (4 °C, overnight) using 2.0% (v/v) paraformaldehyde and 2.5% (v/v) glutaraldehyde in 0.1 M PBS. Cells were washed in PBS, fixed in 1% osmium tetroxide at room temperature (20 °C) for 2 h, and then washed three times in PBS. After that, the cells were dehydrated in a graded acetone series (30%, 50%, 70%, 80%, 85%, 90%, 95%, 100% (v/v) ethanol). The last step was repeated twice for 20 min each time. The specimens were permeabilized at a 2:1 mixture of acetone and Epon overnight at 37 °C, then placed in acetone in Epon (1:1 mixture) for 8 h at 37 °C and finally in pure Epon overnight at 37 °C. Then, the cells were transferred to a casting mould for polymerization at 60 °C. The specimens were sectioned to a thickness of 80 μm (Leica, Wetzlar, Germany). The sections were placed in a mesh copper grid and stained with 2% uranium acetate and lead citrate. Then, the sections were dried overnight at room temperature and examined using a transmission electron microscope (Hitachi, Tokyo, Japan).

### Western blotting

Western blot (WB) analysis was carried out according to previous methods and briefly described as follows. The total protein of cells was harvested with a RIPA lysis solution involving PMSF and phosphatase inhibitors (BioSharp, Hefei, China). Sample proteins were separated by SDS–PAGE and transferred to PVDF membranes. The membrane was blocked for 2 h in 5% skim milk, followed by treatment with primary antibody and secondary antibody. Proteins were visualized with a ChemiDoc MP imaging system (Bio-Rad, USA), and protein expression was analysed with ImageJ software.

### Immunofluorescence staining

Immunofluorescence (IF) staining was carried out according to previous methods and briefly described as follows. CMT-7364 cells and 4T1 cells were cultured in 24-well plates at a density of 1 × 10^5^ cells/mL. After treatment, cells were fixed with 4% (v/v) paraformaldehyde for 30 min and permeabilized with 0.5% Triton X-100 in PBS for 20 min. Subsequently, the cells were blocked with 5% BSA for 2 h and incubated with primary antibodies overnight at 4 °C. FITC-labelled or Cy3-labelled goat anti-rabbit IgG (H + L) was used to incubate the cells in the dark for 1 h and stain with 4,6-diamidino-2-phenylindole (DAPI, Beyotime, China) for nuclear counterstaining for 2 min. In this experiment, the cells were washed three times with PBS between each treatment. Images were observed under a fluorescence microscope (Olympus, Tokyo, Japan).

### In vivo experiments

Six-week-old BALB/c (16–18 g) female mice were purchased from the Hubei Province Experimental Animal Center of Huazhong Agricultural University (Wuhan, China) a week prior to the experiments. The experimental animals were cared for and used under the guidelines of the Institutional Ethical Committee for Animal Care and Use of Huazhong Agricultural University and United States National Institutes of Health. Approximately 5 × 10^7^ 4T1 cells were collected and suspended in 2.5 mL of PBS and then subcutaneously injected into the fourth breast pad of the mice. The mice developed obvious lumps two weeks after the injection. These mice were randomized into five groups and all intraperitoneally injected with different drugs. The mice in the first group were treated with HCQ (50 mg/kg, every other day for 25 days). The mice in the second group were treated with 2-DG (100 mg/kg, every other day for 25 days). The mice in the third group were treated with 1/2 2-DG (50 mg/kg, every other day for 25 days) and HCQ (50 mg/kg, every other day for 25 days) at the same time. The mice in the fourth group were treated with 2-DG (100 mg/kg, every other day for 25 days) and HCQ (50 mg/kg, every other day for 25 days) at the same time. The last group was treated with PBS as the control group. Tumour volume and body weight were measured every 2 days. Tumour volume (*V*) = 0.5 × length × width^2^. Finally, mice were humanely killed at the end of the experiment, tumours were harvested and weighed, and major vital organs, such as the liver, lung, spleen, heart and kidney, from each group were harvested and fixed in 4% paraformaldehyde.

### Mouse tissue section staining

Tumours and organs were subsequently dehydrated and embedded in paraffin 48 h after fixation in formaldehyde and later cut into 5-µm-thick slices for haematoxylin and eosin (H&E) staining. An optical microscope (Olympus, Japan) was used for image collection. The tumour sections were also applied for immunofluorescence staining.

The tumour sections were also applied for immunofluorescence staining. After dewaxed by xylene and gradient ethanol to water, the tumour sections were immersed in citric acid antigen repair buffer (pH 6.0) and heated in a microwave oven for antigen repair. Then, tumour sections were subjected to a similar procedure to step 2.8, including blocking with goat serum, incubating with primary and secondary antibodies, counterstaining with DAPI, and sealed with anti-fluorescence quenching sealing tablets. Images were observed under a fluorescence microscope (Olympus, Tokyo, Japan).

### Statistical analysis

Data are presented as the means ± standard deviation (means ± SD) of at least three independent experiments. The intergroup differences were calculated by two‐way analysis of variance or unpaired Student’s *t* test (GraphPad Prism 8). A level of *p < 0.05 was considered significant, and **p < 0.01, ***p < 0.001, or ****p < 0.0001 was considered extremely significant. A level of *p* > 0.05 (ns) was considered not significant.

## Supplementary information

Supplementary Figure 1

Supplementary Figure 2

Supplementary Figure 3

supplementary legends

Reproducibility checklist

Original Data File-western blot

Original Data File-H&E

Original Data File-H&E

Original Data File-Flow Cytometry

Original Data File-TEM

Original Data File-TEM

Original Data File-Scratch-wound

Original Data File-Cleaved-caspase3-IF

Original Data File-cleaved-caspase3 IF

Original Data File-LC3B-IF

Original Data File-LC3B -IF

## Data Availability

All data are available in the main text or in the supplementary materials.
