# Supplementary figures and images for: The combination of hydroxychloroquine and 2-deoxyglucose enhances apoptosis in breast cancer cells by blocking protective autophagy and sustaining endoplasmic reticulum stress
Source: Cell Death Discov. 2022 Jun 11;8:286. doi: 10.1038/s41420-022-01074-6 (PMC9188615; doi:10.1038/s41420-022-01074-6)

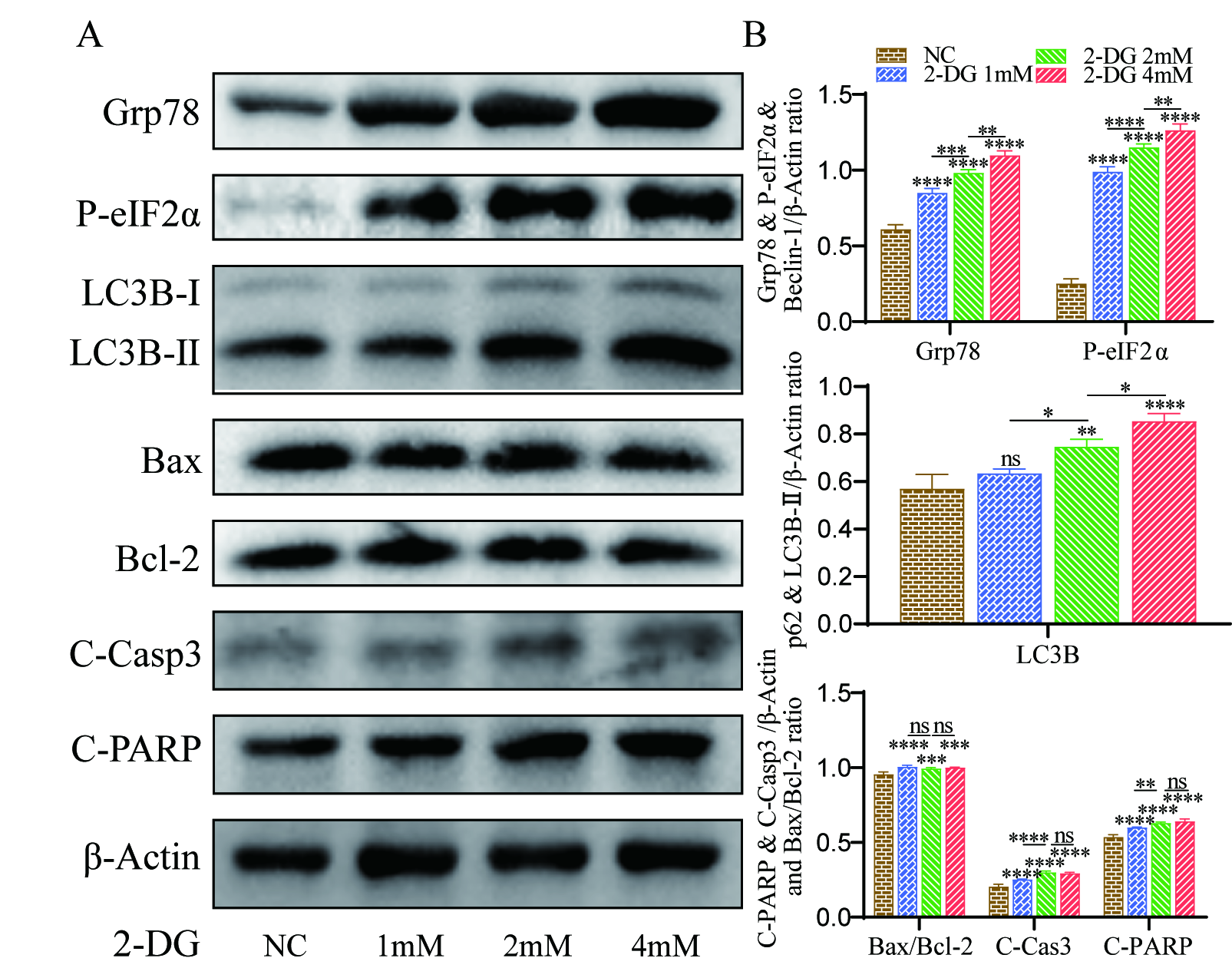

Supplement: Supplementary file 1 — Supplementary Figure 1 [file 41420_2022_1074_MOESM1_ESM.tif]

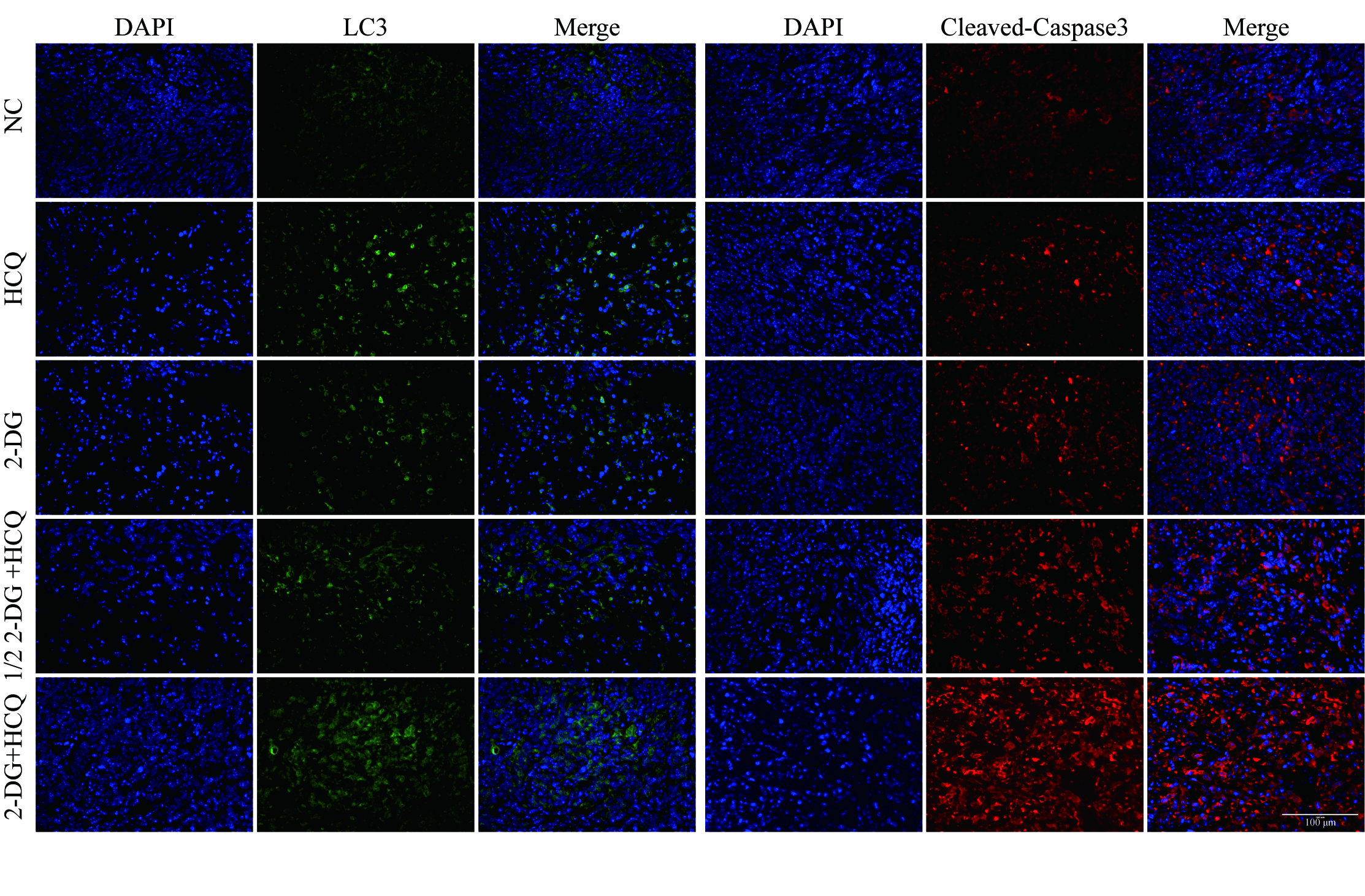

Supplement: Supplementary file 2 — Supplementary Figure 2 [file 41420_2022_1074_MOESM2_ESM.tif]

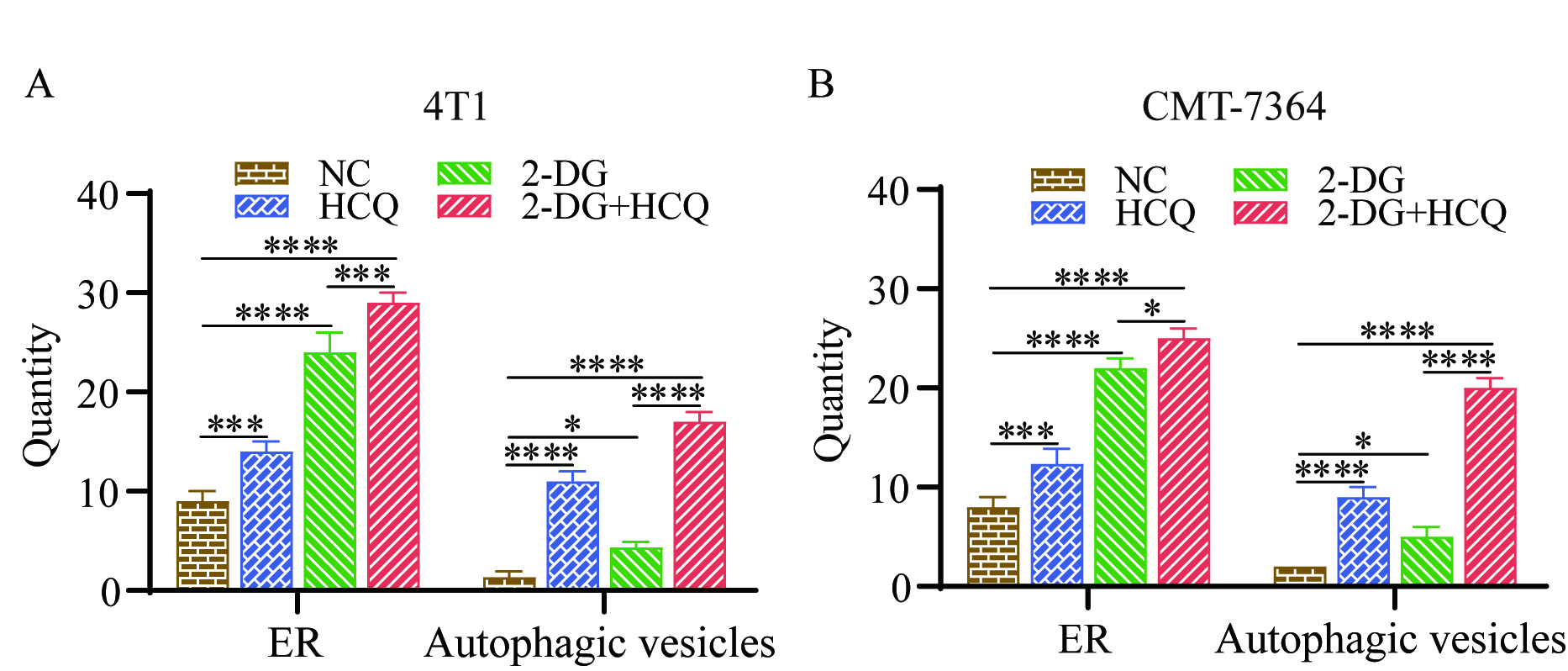

Supplement: Supplementary file 3 — Supplementary Figure 3 [file 41420_2022_1074_MOESM3_ESM.tif]

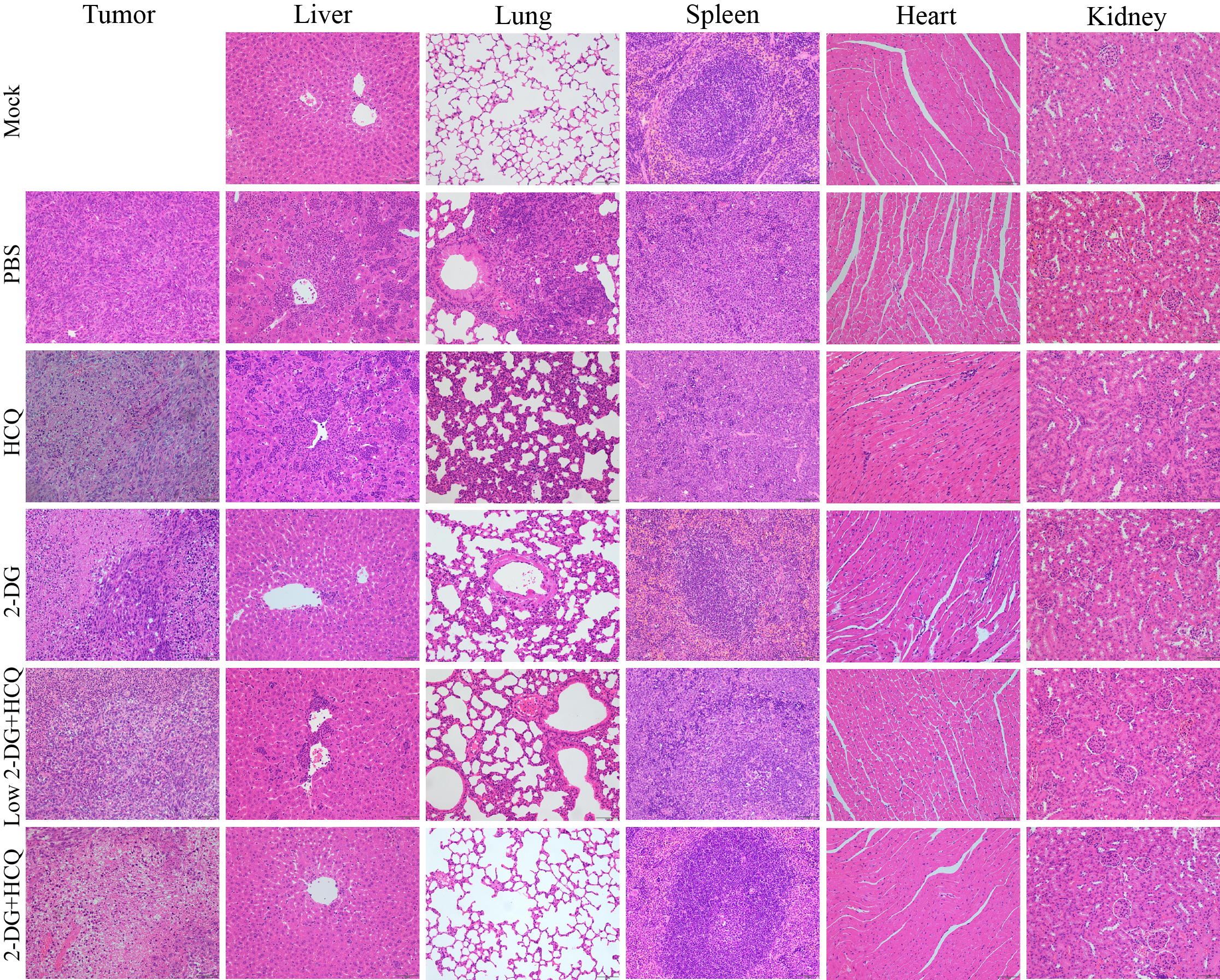

Supplement: Supplementary file 8 — Original Data File-H&E [file 41420_2022_1074_MOESM8_ESM.png]

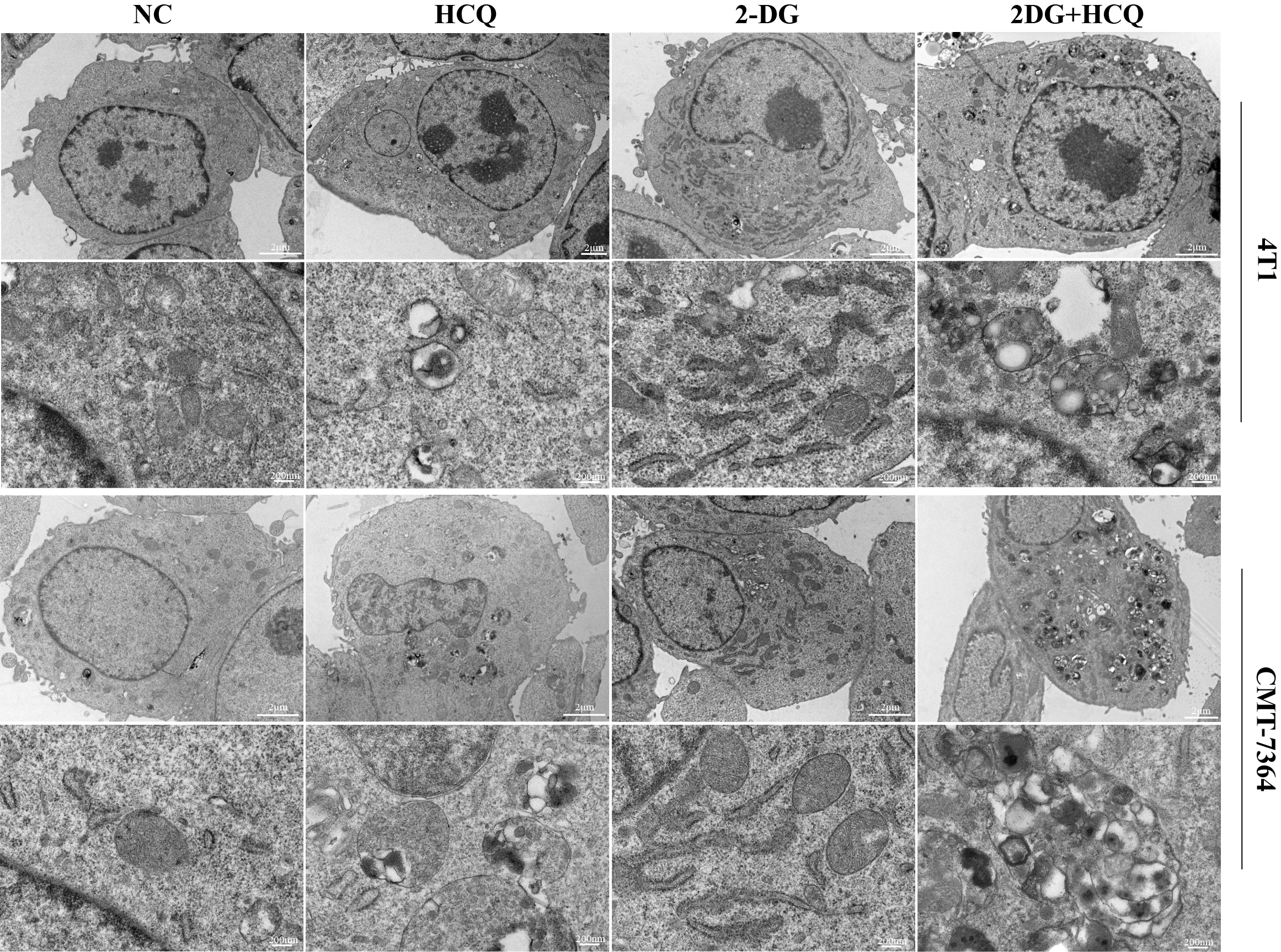

Supplement: Supplementary file 11 — Original Data File-TEM [file 41420_2022_1074_MOESM11_ESM.png]

|     | NC                                                                                  | HCQ                                                                                 | 2DG                                                                                 | 2DG+HCQ                                                                               |          |
|-----|-------------------------------------------------------------------------------------|-------------------------------------------------------------------------------------|-------------------------------------------------------------------------------------|---------------------------------------------------------------------------------------|----------|
| 0h  | 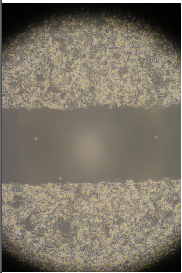   | 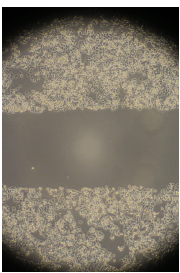   | 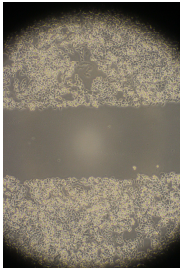   | 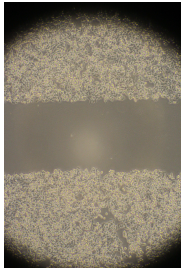   | 4T1      |
| 24h | 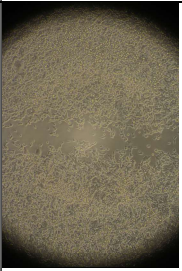   | 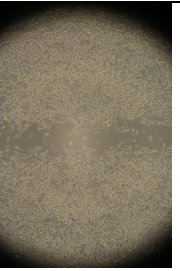   | 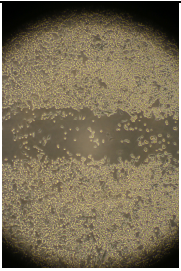   | 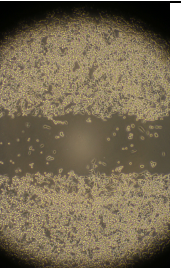   |          |
| 0h  | 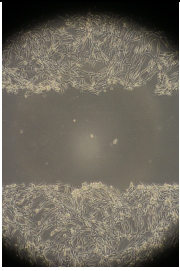  | 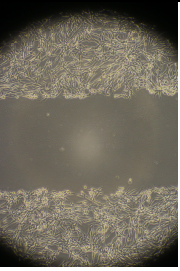  | 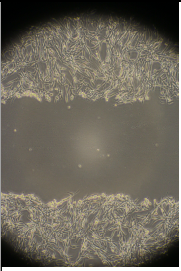  | 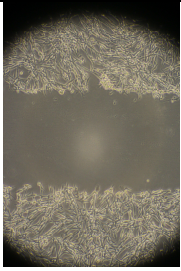  | CMT-7364 |
| 24h | 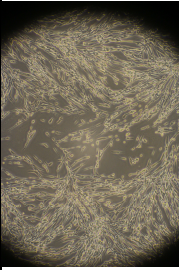 | 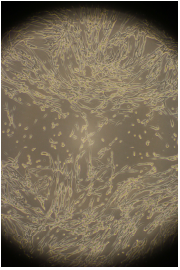 | 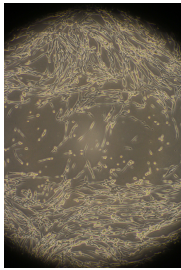 | 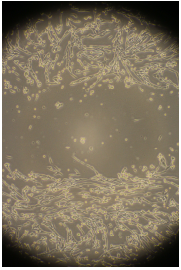 |          |

Supplement: Supplementary file 12 — Original Data File-Scratch-wound [file 41420_2022_1074_MOESM12_ESM.pdf]

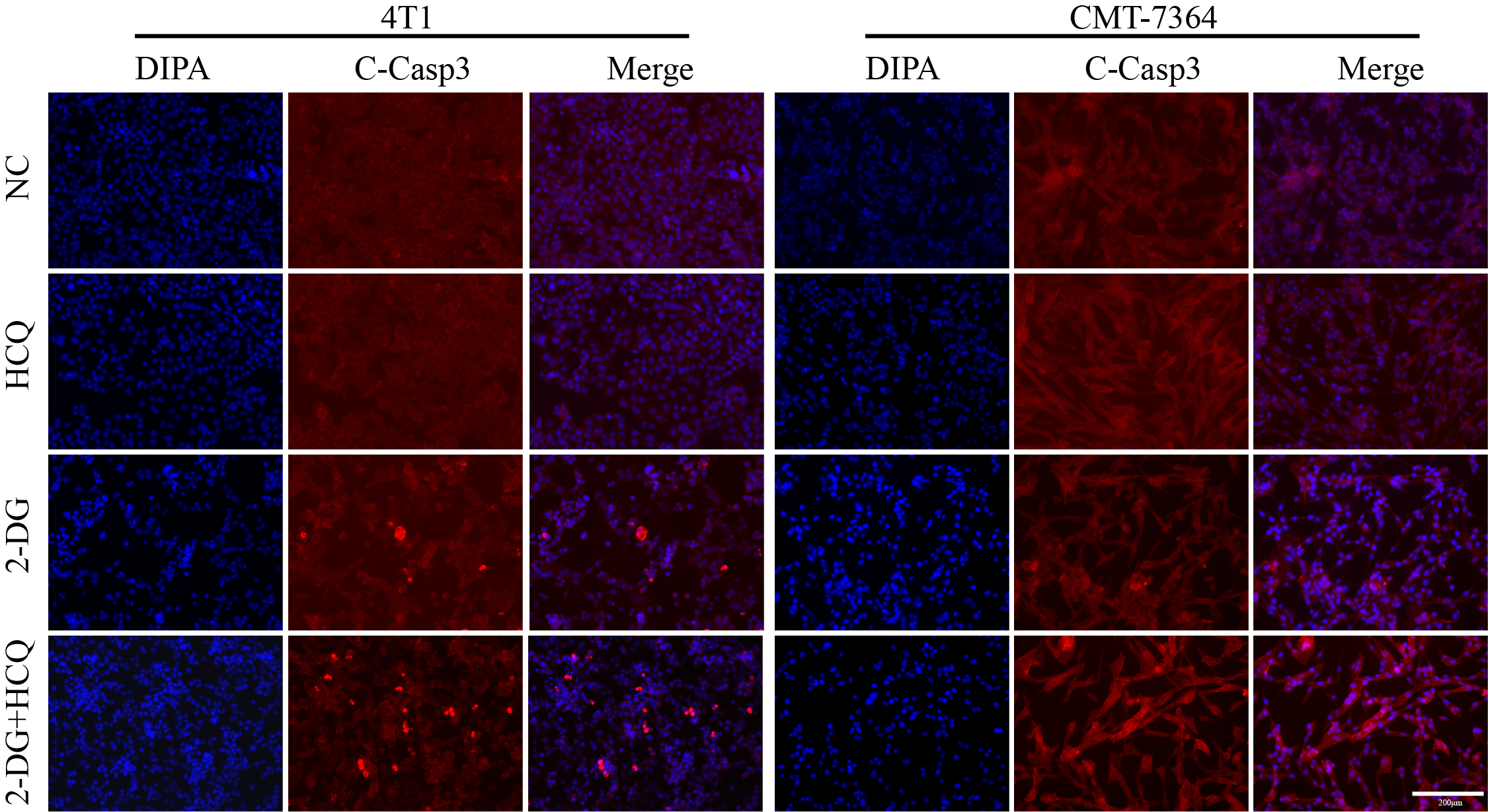

Supplement: Supplementary file 14 — Original Data File-cleaved-caspase3 IF [file 41420_2022_1074_MOESM14_ESM.png]

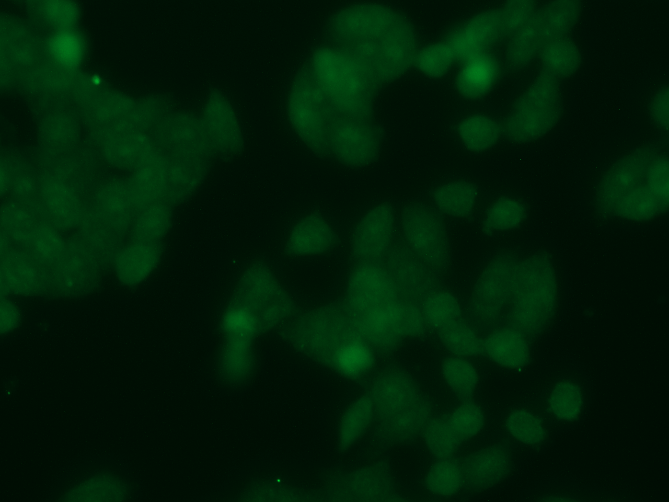
4T1-NC


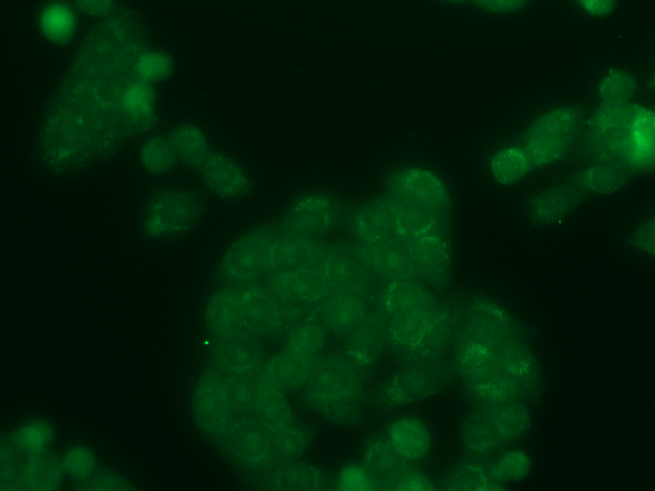


4T1-HCQ


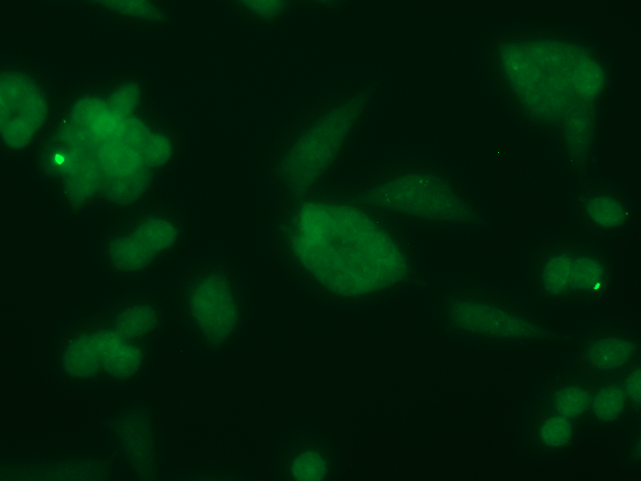


4T1-2DG


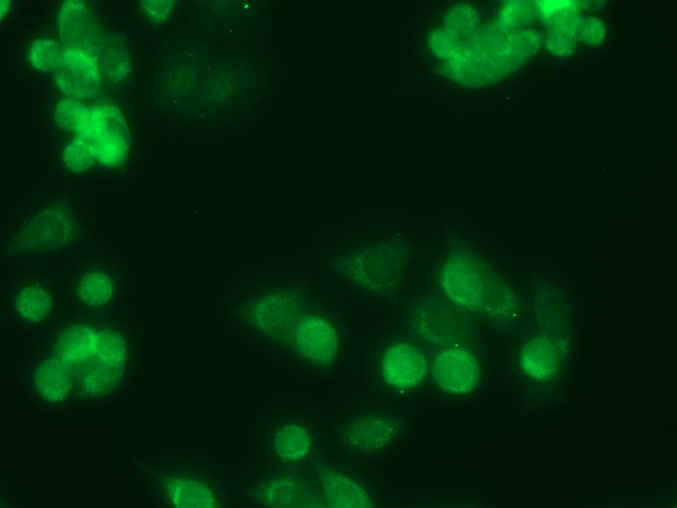
4T1-

HCQ+2DG


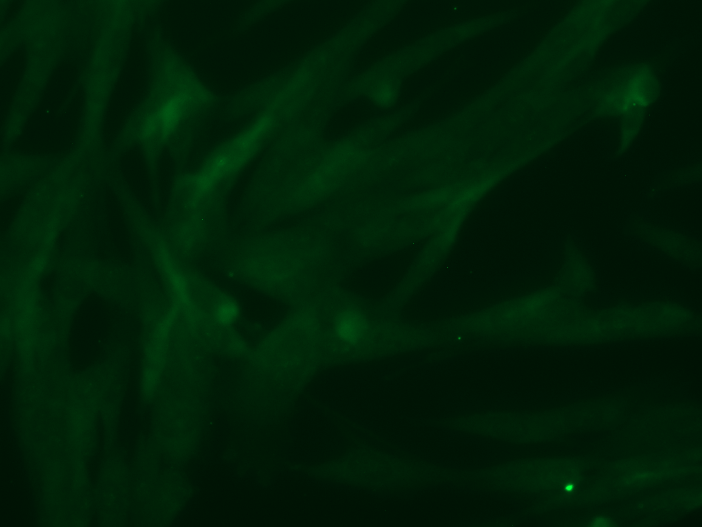
CMT-7364-NC

CMT-7364
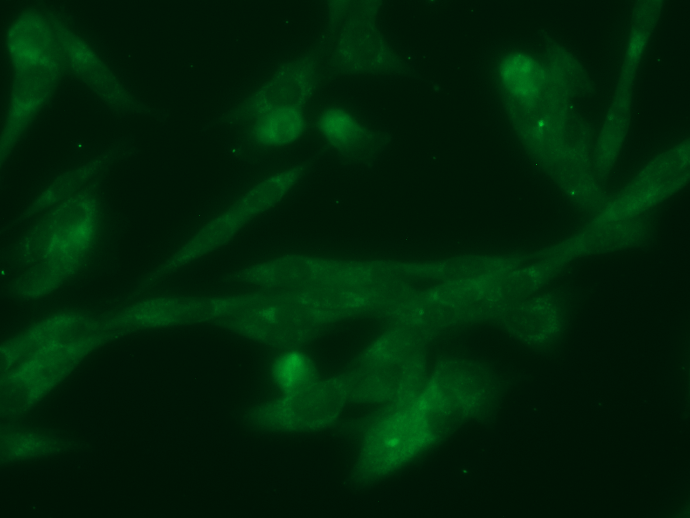
-HCQ

CMT-7364
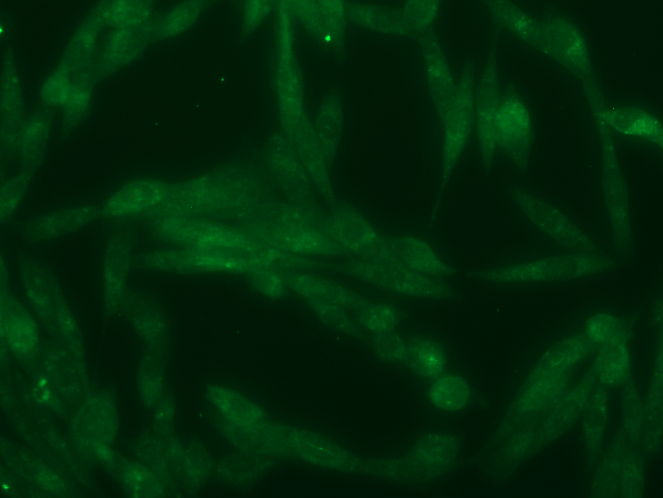
-2DG


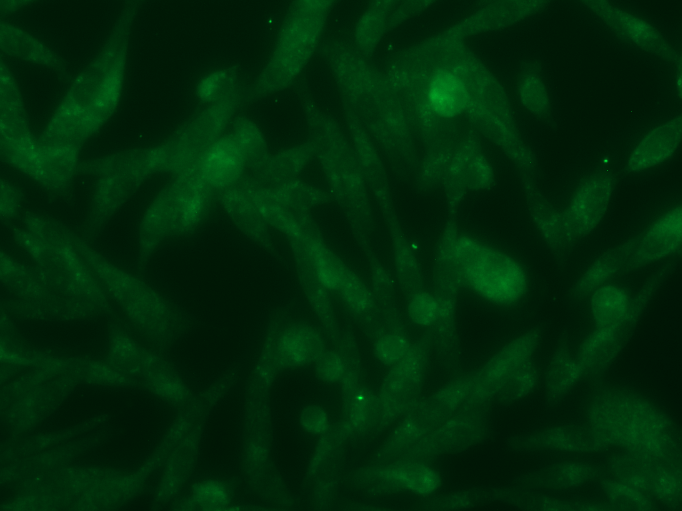


CMT-7364-

2DG+HCQ

Supplement: Supplementary file 15 — Original Data File-LC3B-IF [file 41420_2022_1074_MOESM15_ESM.docx]

4T1-NC

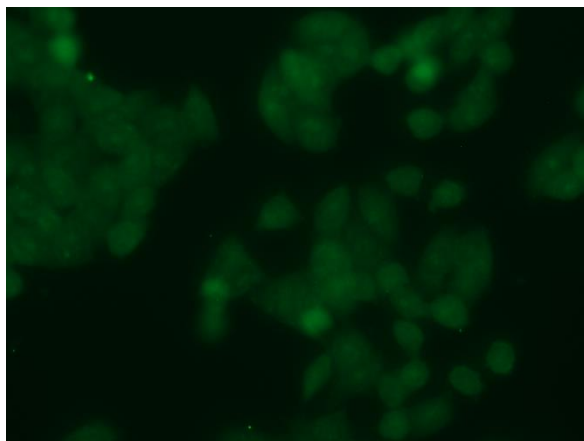

4T1-HCQ

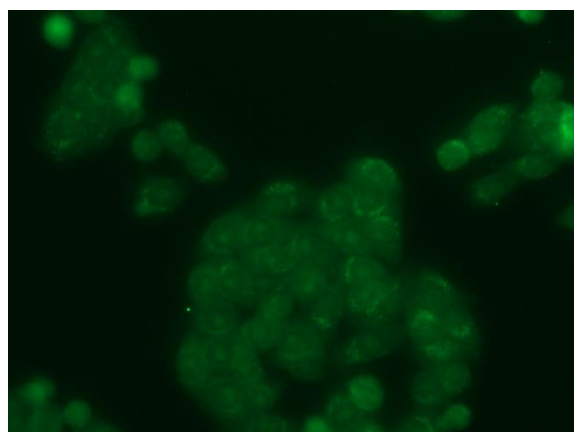

4T1-2DG

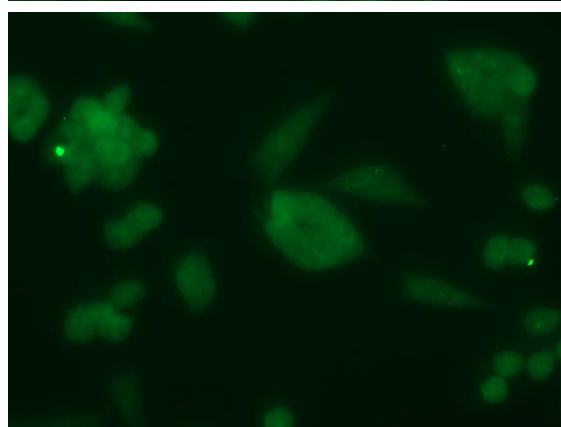

4T1-  
HCQ+2DG

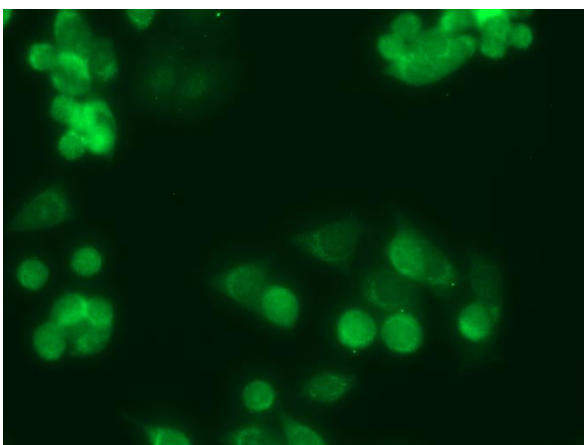

CMT-7364-NC

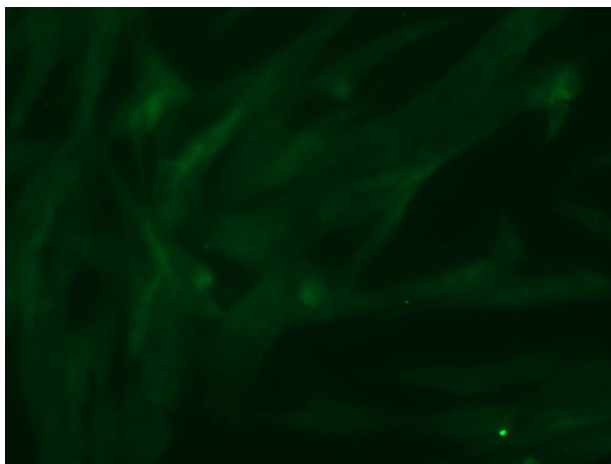

CMT-7364-HCQ

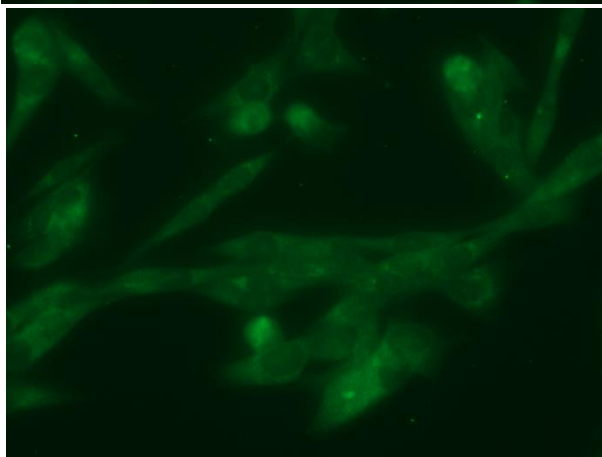

CMT-7364-2DG

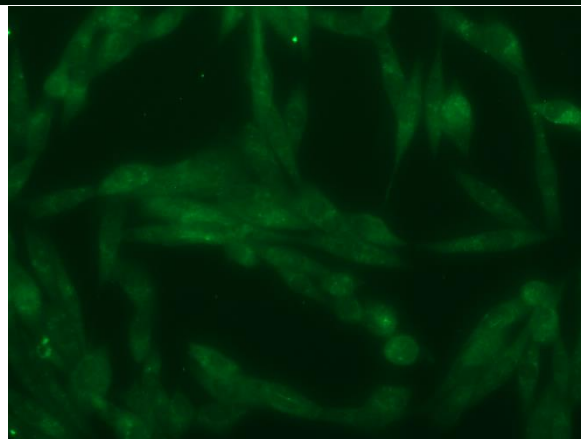

CMT-7364-  
2DG+HCQ

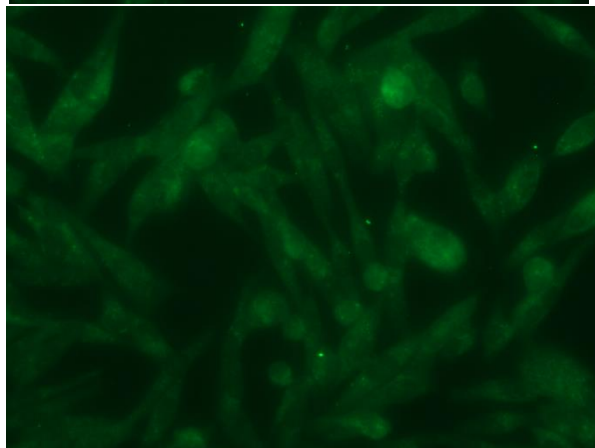

Supplement: Supplementary file 16 — Original Data File-LC3B -IF [file 41420_2022_1074_MOESM16_ESM.pdf]
