# Supplementary material for: The combination of hydroxychloroquine and 2-deoxyglucose enhances apoptosis in breast cancer cells by blocking protective autophagy and sustaining endoplasmic reticulum stress
Source: Cell Death Discov. 2022 Jun 11;8:286. doi: 10.1038/s41420-022-01074-6 (PMC9188615; doi:10.1038/s41420-022-01074-6)
Supplement: Supplementary file 4 — supplementary legends [file 41420_2022_1074_MOESM4_ESM.docx]

**Supplementary Figure S1. The changes of intracellular protein levels in CMT-7964 cells treated with 2-DG alone.**

**A, B** Western blot analyses of Bax, Bcl‐2, cleaved Caspase3 (C-Casp3), and cleaved PARP (C-PARP) from CMT-7364 cells, which were treated with 2-DG for 24 h. β‐actin was used as an internal control. Data are represented as mean ± SD from three independent experiments. *p < 0.05, **p < 0.01, ***p < 0.001, ****p < 0.0001, ns = not significant (p ≥ 0.05).

**Supplementary Figure S2. Immunofluorescence staining of LC3 and Cleaved-Caspase3 in breast tumours treated with HCQ, 2-DG, and their combination.**

Representative immunofluorescence staining for LC3 and Cleaved-Caspase3 in tumours from the xenograft mouse model (400 magnifications, Scale bar 100μm). Blue spots represent cell nuclei, green spots represent LC3‐positive cells and red spots represent Cleaved-Caspase3-positive cells.

**Supplementary Figure S3. Statistical analysis of the number of ER and autophagy vesicles in TNBC cells by TEM detection.**

Statistics and difference analysis of the quantitative changes of ER and autophagic vesicles in 4T1 cells (A) and CMT-7364 cells (B) treated with different treatments in TEM detection. Data are represented as mean ± SD from three independent experiments. *p < 0.05, ***p < 0.001, ****p < 0.0001.
