# Supplementary material for: The combination of hydroxychloroquine and 2-deoxyglucose enhances apoptosis in breast cancer cells by blocking protective autophagy and sustaining endoplasmic reticulum stress
Source: Cell Death Discov. 2022 Jun 11;8:286. doi: 10.1038/s41420-022-01074-6 (PMC9188615; doi:10.1038/s41420-022-01074-6)
Supplement: Supplementary file 6 — Original Data File-western blot [file 41420_2022_1074_MOESM6_ESM.pdf]

Remarks: Some proteins like Bax, Bcl-2, cleaved caspase3 and CHOP, with small molecular mass less than 30 KDa can not be separated on 10% separation gel, but are aggregated on the bromophenol blue band.Therefore, protein markers with sizes of 25, 15 and 10KDa are overlapped in part of the following images

Apoptosis(Fig. 2)

4T1 CMT -7364

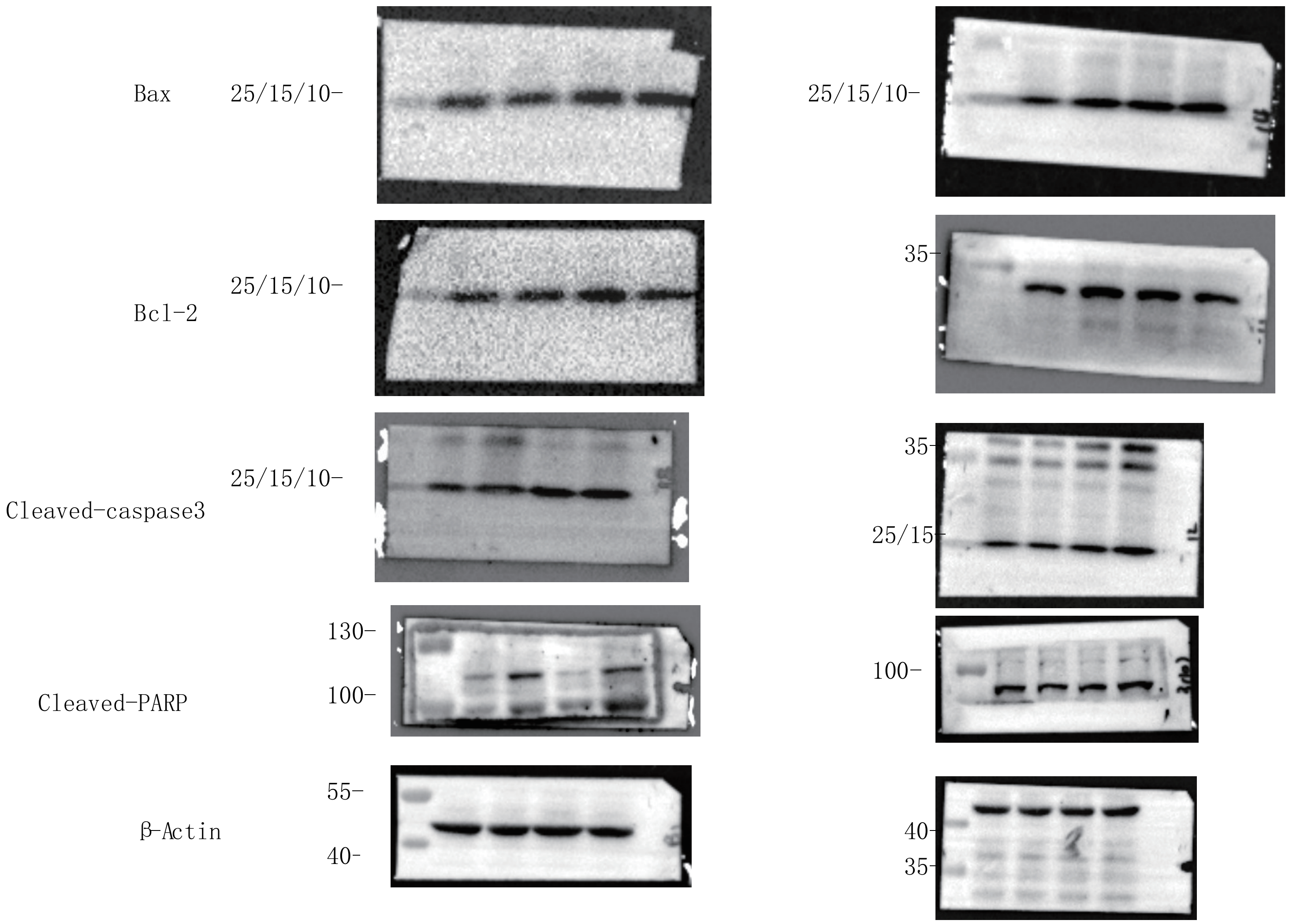

ERS(Fig. 4)

4T1 CMT -7364

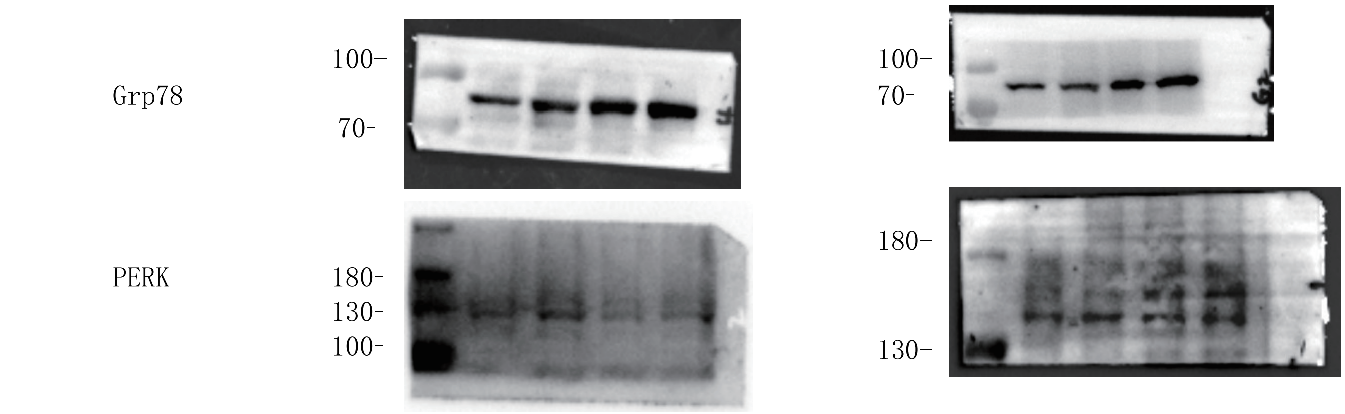

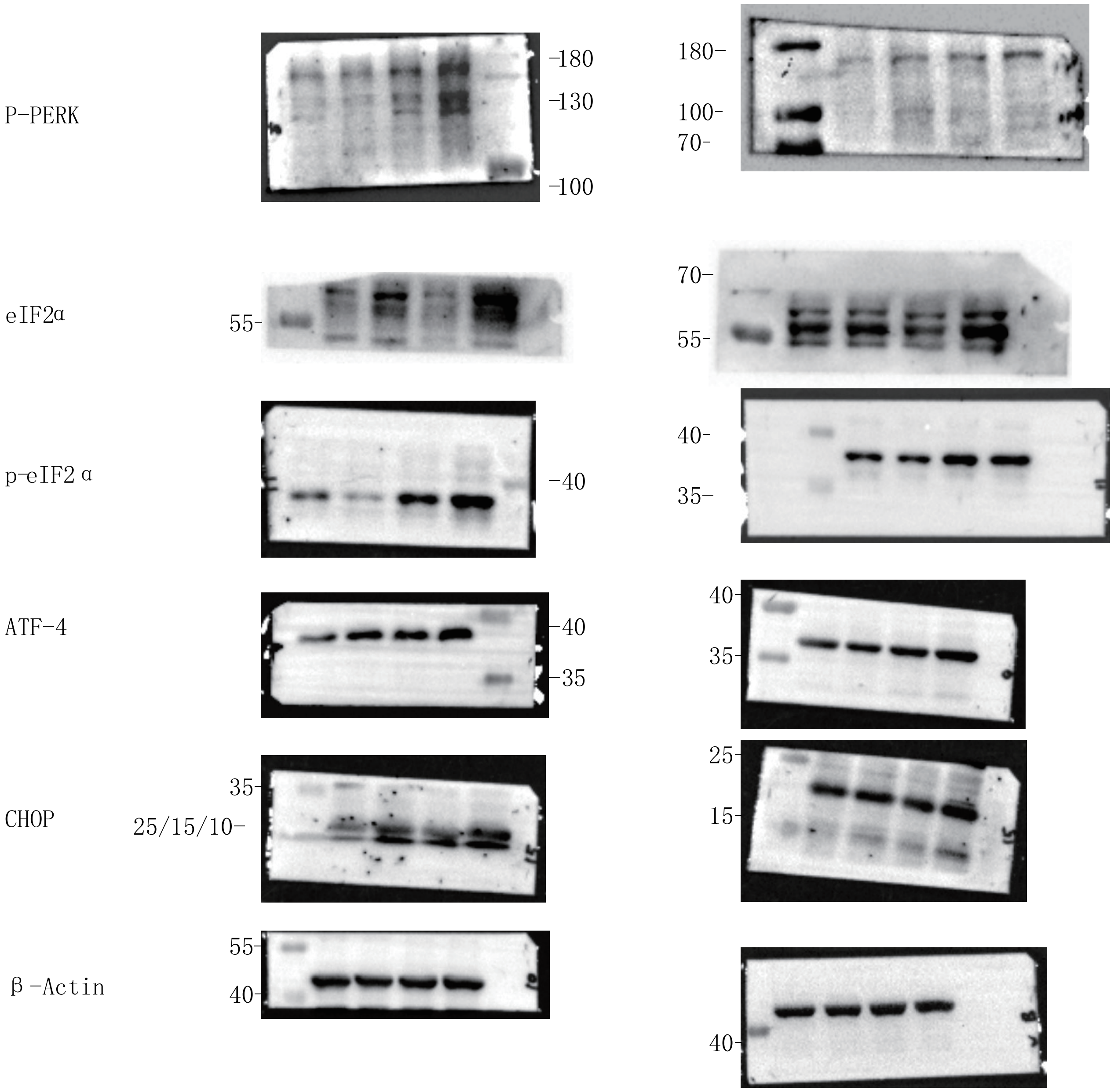

Autophagy (Fig.5)

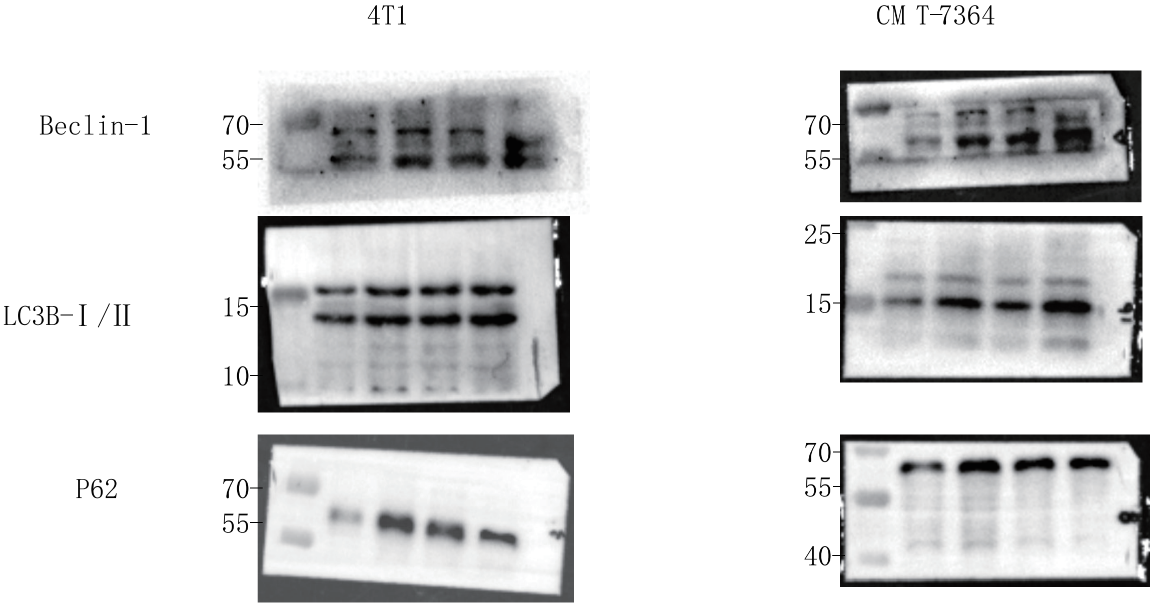

β-Actin

55-
40-
35-

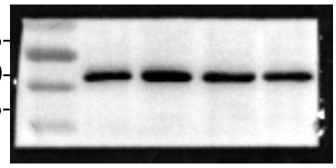

55-
40-

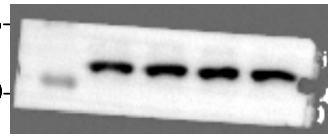

2-DG used alone
（Fig. S1)

CMT-7364

Grp78

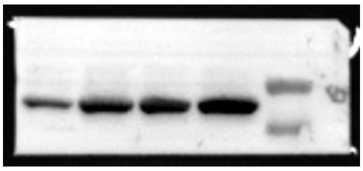

-100
-70

p-eIF2α

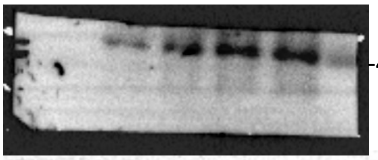

-40

P62

55-
40-

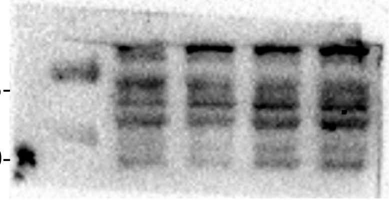

LC3B-Ⅰ/Ⅱ

15-

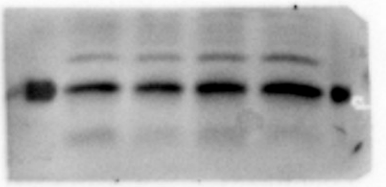

Bax

35-
25/15/10-

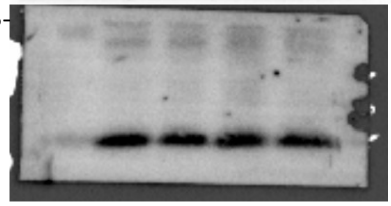

Bcl-2

25/15/10-

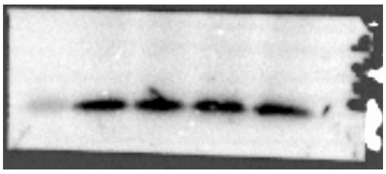

Cleaved-caspase3

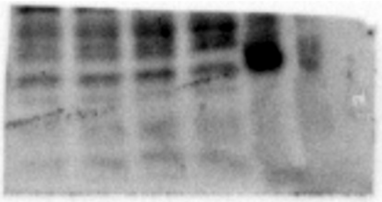

-25/15/10

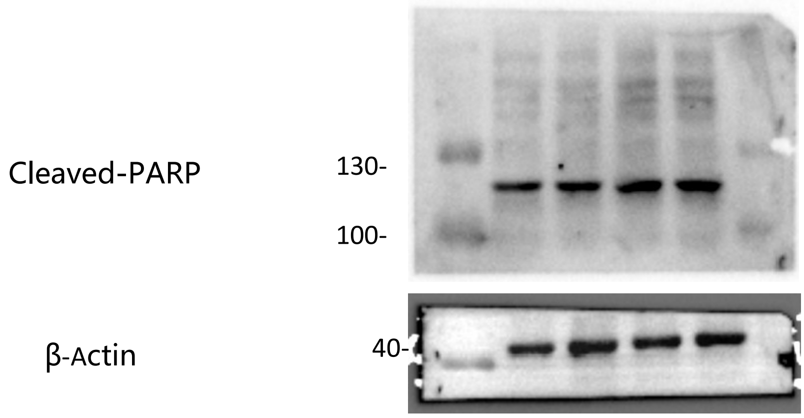
Cleaved-PARP
130-
100-
β-Actin
40-
