# Supplementary material for: The combination of hydroxychloroquine and 2-deoxyglucose enhances apoptosis in breast cancer cells by blocking protective autophagy and sustaining endoplasmic reticulum stress
Source: Cell Death Discov. 2022 Jun 11;8:286. doi: 10.1038/s41420-022-01074-6 (PMC9188615; doi:10.1038/s41420-022-01074-6)
Supplement: Supplementary file 9 — Original Data File-Flow Cytometry [file 41420_2022_1074_MOESM9_ESM.pdf]

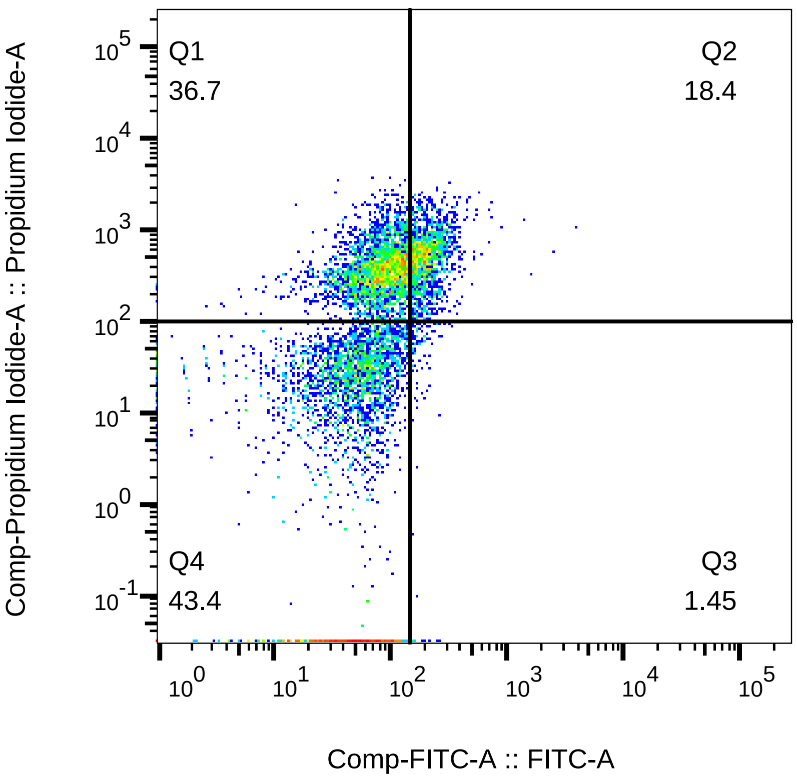

2-DG--4T1.fcs
Lymphocytes
8423

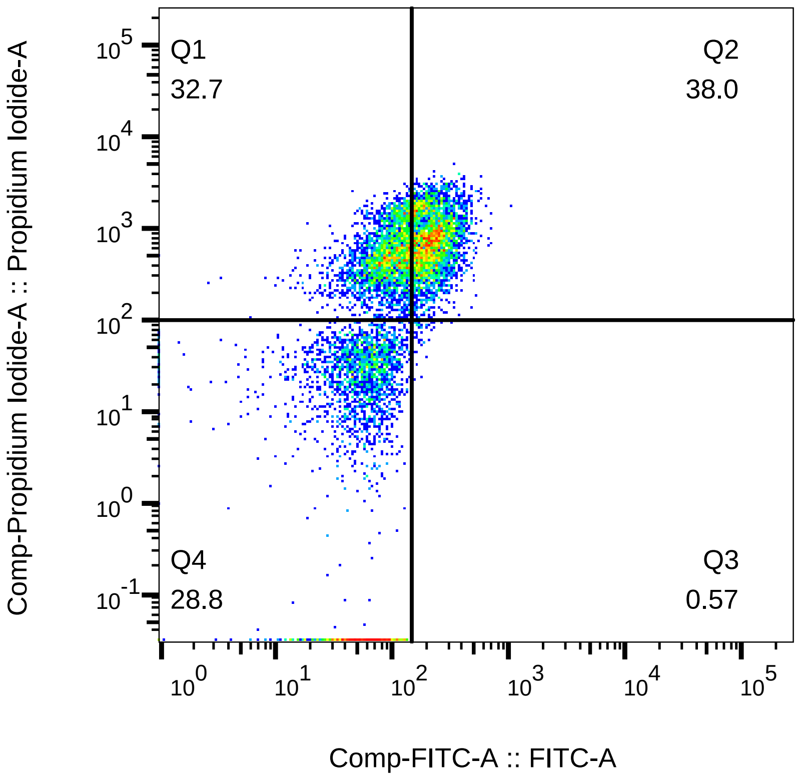

2DG+HCQ--4T1.fcs
Lymphocytes
8792

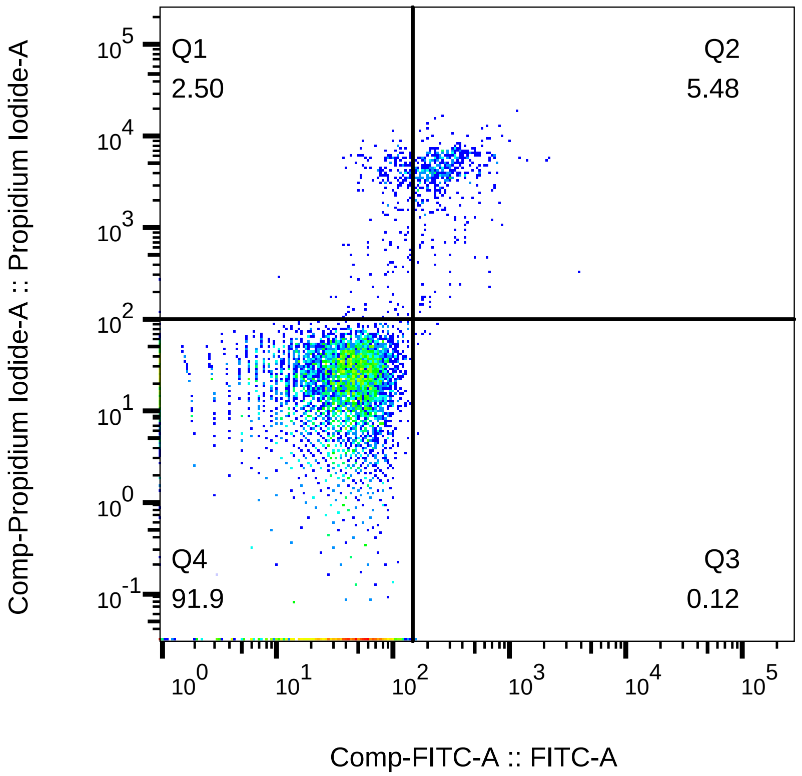

CONTROL--4T1.fcs
Lymphocytes
8631

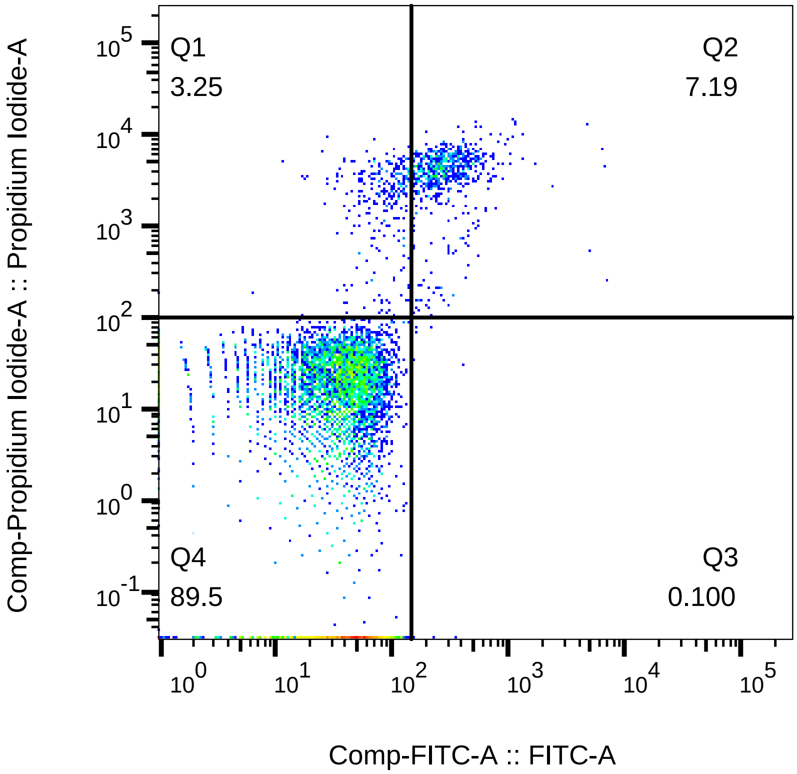

HCQ--4T1.fcs
Lymphocytes
9004

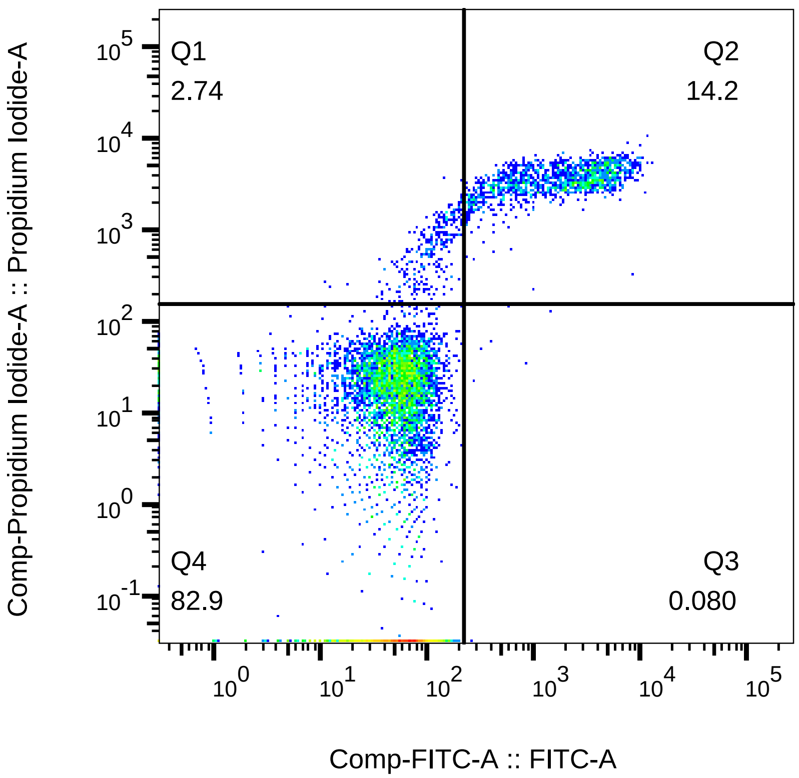

HCQ--7364.fcs
Lymphocytes
9975

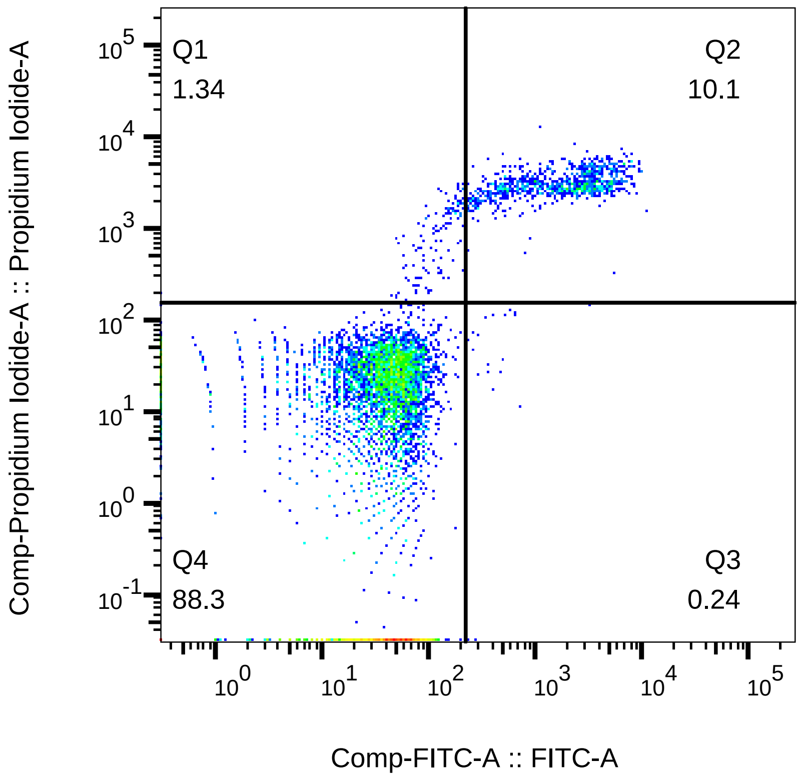

CONTROL--7364.fcs

Lymphocytes

9296

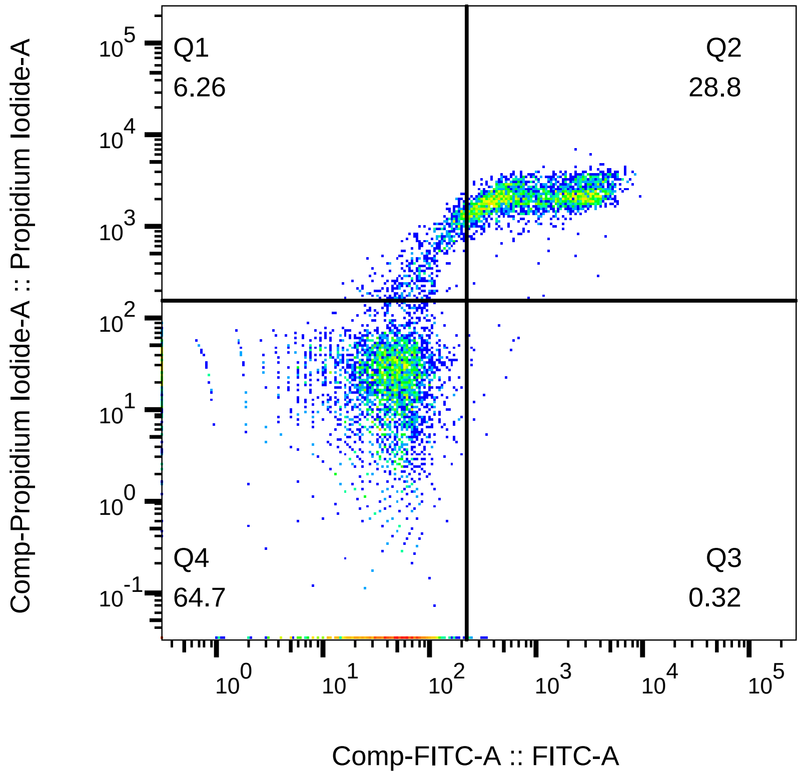

2-DG+HCQ-7364.fcs
Lymphocytes
8782

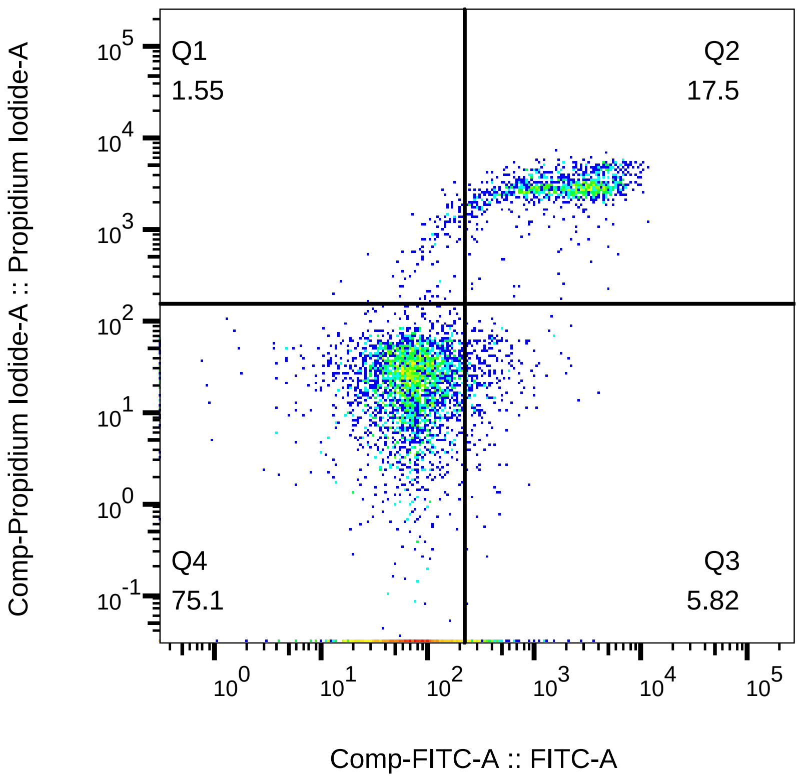

2DG-7364.fcs
Lymphocytes
6198
